# Brain age monotonicity and functional connectivity differences of healthy subjects

**DOI:** 10.1371/journal.pone.0300720

**Published:** 2024-05-30

**Authors:** Siamak K. Sorooshyari

**Affiliations:** Department of Statistics, Stanford University, Stanford, CA, United States of America; New Jersey Institute of Technology, UNITED STATES

## Abstract

Alterations in the brain’s connectivity or the interactions among brain regions have been studied with the aid of resting state (rs)fMRI data attained from large numbers of healthy subjects of various demographics. This has been instrumental in providing insight into how a phenotype as fundamental as age affects the brain. Although machine learning (ML) techniques have already been deployed in such studies, novel questions are investigated in this work. We study whether young brains develop properties that progressively resemble those of aged brains, and if the aging dynamics of older brains provide information about the aging trajectory in young subjects. The degree of a prospective monotonic relationship will be quantified, and hypotheses of brain aging trajectories will be tested via ML. Furthermore, the degree of functional connectivity across the age spectrum of three datasets will be compared at a population level and across sexes. The findings scrutinize similarities and differences among the male and female subjects at greater detail than previously performed.

## Introduction

An understanding of the changes that occur to a brain is fundamental to human neuroscience. While variability in person-to-person brain aging is expected due to genetics, environment, and life events, there are also changes that are more salient. Resting-state fMRI can be collected relatively quickly and easily from subjects of different ages, demographics, and possible pathologies. Atypical brain aging is often reflective of or a precursor to developmental disorders. Furthermore, abnormal trajectories in brain age have been implicated in Parkinson’s disease (PD) [1], Alzheimer’s disease (AD) [2], as well as several other neural pathologies [3] and even mortality [4]. Utilizing fMRI data to investigate brain aging dynamics entails examining alterations in measures of functional connectivity (FC). Typically, patterns of correlated activity between regions of interest (ROIs) are compared between conditions to assess whether there are changes. In the case of aging, differences between connectivity maps are quantified and used to predict age with the purpose of identifying if and how brain signals change over time. Due to its importance, age prediction from brain activity has been undertaken across sizable datasets.

Subsequent to correlational studies and graph-theoretic approaches, machine learning (ML) has been used to discover structure by performing either regression or classification. There exists a growing number of works that use ML to answer questions pertaining to brain aging. In [5] support vector regression (SVR) was used to estimate brain age. Three groups of subjects were considered with the ages of a subject lying in between 6 and 35 years. The considered age range was limited in comparison to more recent works as well as the data considered in this work. The work considered 12,720 FC features per subject and pare to 200 for the analysis. LASSO regression was used in [6] to predict brain age by considering over 14,000 healthy participants from the UK Biobank. Interestingly, that work found that resting and task-based fMRI are relatively uninformative in comparison to several variants of MRI, i.e. T1-weighted MRI, T2-FLAIR,T2*, and diffusion-MRI, at chronological age prediction. A review on the use of ML techniques for rsfMRI analysis is provided in [7]. We shall highlight several of the more recent and particularly relevant works below.

The deviation of a person’s brain age from biological age may also be affected by sex as there is no reason to assume that the prospective changes are uniform among males and females. Works are increasingly incorporating sex as either a predictor or a response variable when evaluating brain age. A recent study [8] considered over 4,000 subjects across a wide age spectrum from five different sites. Ridge regression was used, and brain age prediction performance was quantified via the mean absolute error (MAE) and correlation between the predicted and actual ages. The work quantified the effects of a multiscale study by considering various numbers of functional networks in the predictive analysis. In [9] the authors consider over 9,000 different pipelines consisting of hyperparameter values across a large (over 14,000) number of subjects from the Human Connectome Project (HCP) and UK Biobank. Correlation-based measures of functional connectivity were computed and used as inputs for several classifiers including variants of deep learning (DL). It was noted that the regularized regression method elastic net performs comparably with DL schemes for age prediction.

We use an ML pipeline to study whether brain aging is a progressive phenomenon that can be characterized by a monotonic relationship. Recordings of different age groups will be used in a training procedure with the resulting machine asked to classify subjects from age groups that the machine has not previously encountered. The findings will reveal a prospective similarity or difference among the connectivity of brains from a test set to those that are younger or older. Several hypotheses will be presented and tested. Namely, what degree of monotonicity exists in the changes experienced by brains during aging? How consistent are the findings across datasets? Does the aging trajectory of young brains provide insight into the aging trajectory of older brains? Conversely, are alterations in older subjects informative of brain activity earlier in life? To the best of our knowledge, such analysis has not been previously undertaken in imaging studies that assess FC.

The dedifferentiation theory of aging was presented to reflect evidence of brain activity that is localized to specific regions in young adults becoming less localized with aging [10]. While there exist advancements to and affirmations of the dedifferentiation theory of aging [11, 12], we scrutinize the variability in the FC that appears at the various age groups by computing the number of significant connections across the brains of subjects to evaluate how they are altered by aging. The study shall be undertaken separately for male and female subjects as well to assess sex differences. The analysis constitutes novel findings and quantifies the modularity with the inclusion of anticorrelations. The majority of FC fMRI works restrict attention to positive connections and do not differentiate by sex.

## Materials and methods

### Subjects, imaging acquisition, and ROI definition

Three different datasets were considered. Table 1 contains a comparative listing of the number of subjects per age group in each dataset. The age groups have been selected on (approximately) a decade basis. A subject division by sex is also provided and will be necessary for the assessment of FC differences among males and females during aging. The first dataset consists of participants from the International Neuroimaging Data-sharing Initiative (INDI), 1000 Functional Connectomes’ Project (1000FCP) [13, 14]. The cohort contains N = 887 individuals from 17 centers with the requirement that a center provide a minimum of 10 participants with full brain anatomical and functional coverage. The ages of the subjects spanned 21–85 years, and 514 were female. All subjects were at rest and not performing any task during recording. Some subjects had their eyes open while some had their eyes closed, and each recording consisted of signals collected for 42 ROIs. Our analysis is based upon preprocessed data of ROIs obtained from a previous study. We provide an overview of the most relevant section from the methods of [11]. Seed-based analysis was performed as previously described [15, 16] to identify seven networks. The following seed regions were used to delineate the networks: left posterior cingulate cortex, right frontoinsula, right intraparietal sulcus, right superior parietal cortex, left auditory cortex, right visual cortex, and left motor cortex. Each seed was defined as a 6 mm radius sphere centered on previously published foci. Correlation maps were produced by extracting the time course from each of the above seeds. Then, the Pearson correlation coefficient (PCC) between the time course and the time course of each voxel across the whole brain was computed to create the voxel-wise connectivity maps. The maps from a young age group (N = 458, 21–30 years) were used to identify peak coordinates of additional ROIs representing each network. All regions were defined as 6 mm radius spheres around the peak coordinate. Information regarding the participants, number of time points collected for each recording, and the rsfMRI acquisition and epidemiological parameters of the different centers are detailed in Table 2 as well as Tables 1 and 2 of [11].

**Table 1:** An itemization of the number of subjects per age group associated with rsfMRI data via the 1000FCP (N = 887), NKI-RS recording center of 1000FCP (N = 307), SRPBS (N = 709), and camCAN (N = 652) datasets. The number of subjects per age group are bifurcated by sex via the convention (male, female). *The 1000FCP subjects in Group #1 were, more precisely, in the 21–30 range.

Group #	Age group (years)	1000FCP, # of subjects	SRPBS, # of subjects	camCAN, # of subjects	NKI-RS (1000FCP), # of subjects
1	18–30	458 (215, 243)*	327 (227, 100)	79 (35, 44)	65 (37, 28)*
2	31–40	85 (44, 41)	126 (81, 45)	105 (56, 49)	27 (8, 19)
3	41–50	119 (40, 79)	115 (48, 67)	101 (43, 58)	71 (16, 55)
4	51–60	119 (38, 81)	69 (29, 40)	101 (54, 47)	67 (12, 55)
5	61–70	67 (22, 45)	61 (30, 31)	104 (56, 48)	45 (13, 32)
6	71–80	-	-	117 (55, 62)	-
6	71+	39 (14, 25)	11 (8, 3)	-	32 (11, 21)
7	81+	-	-	45 (23, 22)	-

**Table 2:** (Top) Recording center demographics for the N = 887 subjects from the 1000FCP that were used in the analysis. Additional details such as the number of slices, voxel size, and subject handedness can be found in Table 1 of [11]. (Middle) Recording center demographics for the N = 709 subjects from SRPBS, with additional information available in Table 5 of [17]. (Bottom) Information on the N = 652 subjects from the camCAN dataset that consisted of a single recording center. Further details about the subjects and study can be found in [18, 19].

Recording center	Subjects (male, female)	Age (years)	Scanner, TR (s)	Time points	Eyes
NKI-RS	307 (97, 210)	21–85	3T, 2.5	120	Open
Beijing	119 (48, 71)	21–26	3T, 2	225	Closed
Cambridge	101 (40, 61)	21–30	3T, 3	119	Open
COBRE	66 (47, 19)	21–65	3T, 2	150	NA
Milwaukee	43 (14, 29)	44–65	3T, 2	175	NA
New York	32 (18, 14)	22–49	3T, 2	192	Open
St. Louis	31 (14, 17)	21–29	3T, 2.5	127	Open
Atlanta	28 (13, 15)	22–57	3T, 2	205	Open
Berlin	26 (13, 13)	23–44	3T, 2.3	195	Open
Cleveland	26 (9, 17)	24–60	3T, 2.8	127	Closed
Dallas	21 (10, 11)	21–71	3T, 2	115	NA
Queensland	18 (11, 7)	21–34	3T, 2.1	190	Open
Orangeburg	17 (13, 4)	25–55	1.5T, 2	165	Closed
Palo Alto	17 (2, 15)	22–46	3T, 2	245	NA
Munich	14 (9, 5)	63–73	1.5T, 3	72	Closed
Leiden 1	10 (10, 0)	21–27	3T, 2.18	215	Closed
Leiden 2	11 (5, 6)	21–28	3T, 2.2	215	Closed
Recording center	Subjects (male, female)	Age (years)	Scanner, TR (s)	Time points	Eyes
Kyoto	234 (141, 93)	18–78	3T, 2.5	240	Open
ATR	108 (88, 20)	20–30	3T, 2.5	240	Open
Osaka	29 (21, 8)	29–73	3T, 2.5	240	Open
SWA	101 (86, 15)	19–55	3T, 2.5	244	Open
Hiroshima	237 (87, 150)	20–79	3T, 2	143	Open
Recording center	Subjects (male, female)	Age (years)	Scanner, TR (s)	Time points	Eyes
camCAN	652 (322, 330)	18–88	3T, 1.97	261	Closed

The rsfMRI data from N = 709 subjects (286 female) spanning ages 18–79 years in the Japanese Strategic Research Program for the Promotion of Brain Science (SRPBS) project [17] was also used. Healthy control (HC) data was taken from five sites in Japan: Osaka, Showa, Kyoto, and Hiroshima Universities as well as the Advanced Telecommunications Research Institute (ATR). Each recording consisted of signals collected at 140 ROIs of a HC. The voxel sizes were generally not the same across the five sites, however, the values were similar by being between 3 mm x 3 mm x 3 mm and 4 mm x 4 mm x 4 mm (please see Table 5 of [17]). A scanning time of 10 minutes was used, and the subjects had their eyes open and fixated on a point during the recording. The 9730 unique PCC values were provided as part of the dataset, thus no additional processing was required to attain the features. The acquisition and imaging parameters for SRPBS are shown in Table 2 and in more detail via Table 5 and 9 of [17].

Lastly, we used rsfMRI data that was collected at one recording center as part of the Cambridge Centre for Aging and Neuroscience (CamCAN) study [18, 19]. A single testing center provided N = 652 subjects (330 female) spanning ages 18–88 years. The subjects had their eyes closed during the recordings and followed the instruction to not think of any one thing in particular. A voxel size of 3 mm x 3 mm x 4.44 mm was used with an acquisition time of 8 minutes and 40 seconds. The acquisition and imaging parameters are further detailed in Table 2 of [19]. To compute the FC from ROI recordings, the Craddock unsmooth parcellation was used to attain 840 ROIs. The mean intensity across voxels was used. Although the parcellation provided 840 ROIs, we considered 808 across each subject as 32 had missing values for some of the subjects. The PCC values were computed by correlating the time courses of the ROIs.

### Preprocessing and head motion correction

With the 1000FCP dataset, the functional images were preprocessed in [11] using the FSL software (FMRIB Software Library v. 5.0.1, Oxford, UK) and SPM software (Statistical Parametric Mapping software package, Wellcome Department of Imaging Neuroscience, London, UK) following conventional methods originally described in [16, 20]. The following steps were preformed: 1) slice-timing correction; 2) rigid-body motion correction; 3) registration to the MNI152 space; 4) regression of nuisance variables including ventricles, white matter (WM), and global average signal 5) temporal bandpass filtering (0.01–0.08 Hz). The preprocessing included rigid body correction for motion within and across runs, normalization to the standard echo-planar imaging (EPI) template of the Montreal Neurological Institute (MNI), and compensation for slice-dependent time shifts. Several key acquisition parameters are shown in Table 2 with additional information such as the number of slices, voxel size, and subject handedness available in Table 1 of [11]. The preprocessed functional data (in atlas space) were temporally filtered to remove constant offsets and linear trends over each run while retaining frequencies below 0.08 Hz. Data were spatially smoothed using a 4 mm full-width half-maximum (FWHM) Gaussian blur. Sources of spurious or regionally non-specific variance were removed by regression of nuisance variables. This included six parameters obtained by rigid body head motion correction, the signal averaged over the whole brain (global signal), the signal averaged over the lateral ventricles, and the signal averaged over a region centered in the deep cerebral WM. Temporally shifted versions of these waveforms were removed by inclusion of the first temporal derivatives (computed by backward differences) in the linear model. The analysis used via [11] to account for head motion is summarized as follows. The parameters were calculated for each participant as proposed by [22]. Framewise displacement (FD), which represents head displacement from volume to volume, was computed as the sum of the first derivative of the six rigid-body motion parameters estimated during standard volume realignment. Delta variation signal (DVARS), which represents the change in BOLD signal intensity from one frame to the next, was computed as the root mean square average of the first derivative of fMRI signals across the entire brain. A standardized version of DVARS was applied according to [21]. Volumes with FD value over 0.5 mm or DVARS value over 1.5 IQRs above the 75th percentile were removed with one prior and two subsequent volumes. Sixteen individuals from several centers were excluded due to excessive head motion during rsfMRI acquisition according to the described movement parameters criteria [22].

The preprocessing of the SRPBS rsfMRI data are detailed in [17]. This was primarily done using SPM8 implemented in MATLAB (R2016b; Mathworks, Natick, MA). The first 10 seconds of data were discarded to allow for T1 equilibration. Preprocessing steps included slice-timing correction, realignment, co-registration, segmentation of T1-weighted structural images, normalization to MNI space, and spatial smoothing with an isotropic Gaussian kernel of 6 mm FWHM. The ROIs were delineated according to 140 regions defined by the Brainvisa Sulci Atlas and three subregions of the cerebellum (the left and right cerebellum, and the vermis). The BOLD signal time courses were extracted from the ROIs, and a bandpass filter (0.008–0.1 Hz) was applied to the time courses before a regression procedure. Filtered time courses were linearly regressed on temporal fluctuations of the WM, the cerebrospinal fluid (CSF), and the entire brain, as well as six head motion parameters. Fluctuation in each tissue class was determined from the average time course of the voxels within a mask created by segmentation of the T1 image. Extracted time courses were then bandpass filtered (0.008–0.1 Hz) before linear regression, as was done for regional time courses. Then, for each individual, a matrix of 9,730 connections between the 140 ROIs was calculated by evaluating PCCs of BOLD signal time courses. The flagged frames in the previous procedure were discarded. After calculating the FD, volumes with FD > 0.5 mm were removed. Further details and information about the procedure to calculate the matrix are outlined in [17, 21].

The camCAN preprocessing of the rsfMRI data was performed and is detailed in [19]. The procedure is summarized as follows. Co-registered T1 and T2 images were used in a multi-channel segmentation (SPM12 Segment, based on “New Segment” in SPM8 [23]) routine in order to extract probabilistic maps of six tissue classes: gray matter (GM), WM, CSF, bone, soft tissue, and residual noise. Images from each subject were coregistered to the subject’s T1-weighted image using a rigid-body (6-df) linear transformation. Normalization parameters from the diffeomorphic anatomical registration through exponentiated lie algebra (DARTEL) procedure [24] were applied. The native-space GM and WM images for all participants who passed quality-control checks were submitted to diffeomorphic registration to create group template images. The group template was then normalized to the MNI template via an affine transformation. Then combined normalization parameters were applied to each individual participant’s GM and WM images. Individual normalized GM and WM images were smoothed (8 mm FWHM Gaussian kernel). Data from each recording was unwarped to compensate for magnetic field inhomogeneities, then realigned to correct for motion, and slice-time corrected. The EPI data were co-registered to the T1 image, and the normalization parameters were applied to warp functional images into MNI space. The mean regional time-courses were then extracted using the template method.

### Computed features and description of ML pipeline

**Fig 1:**
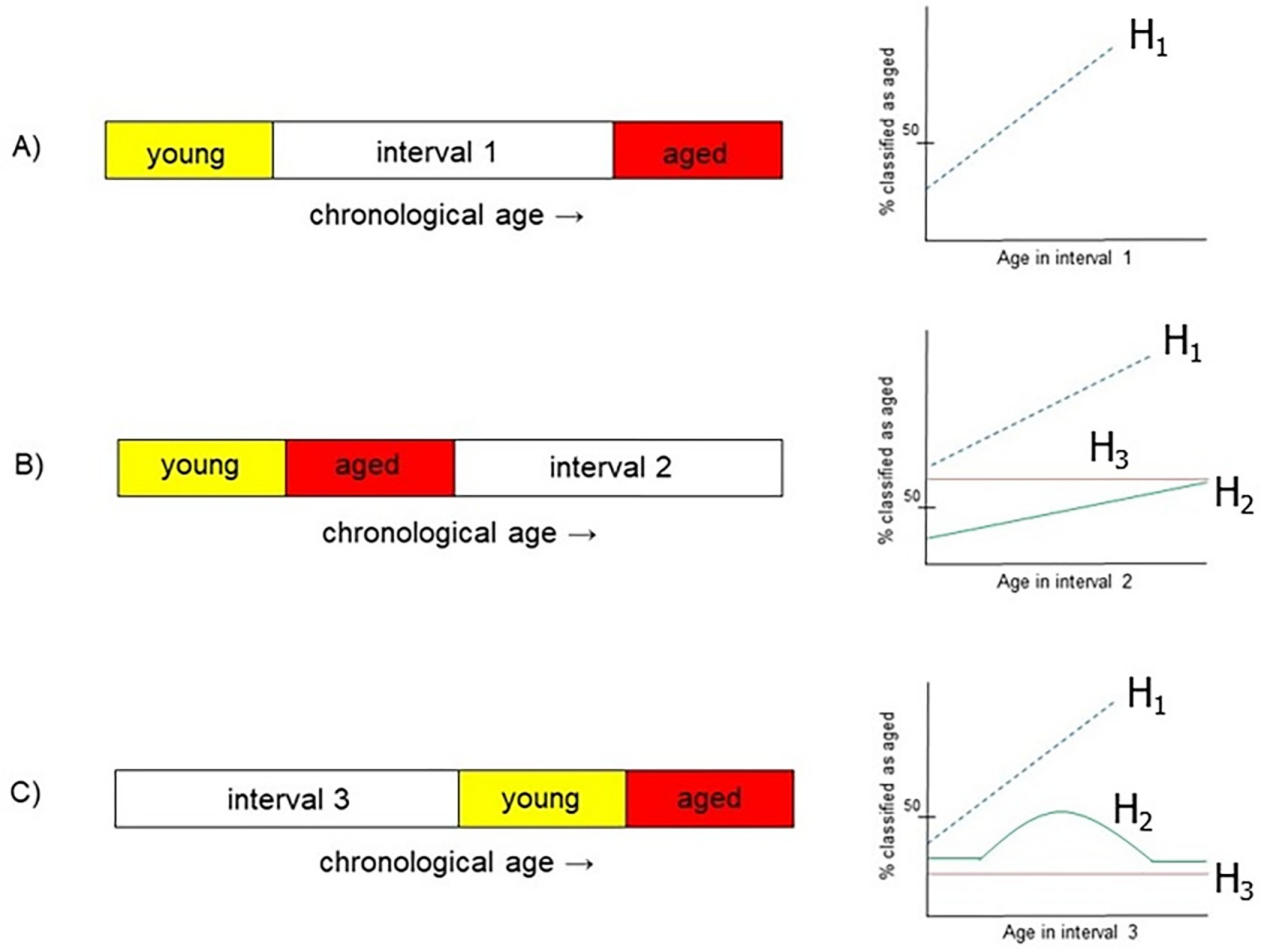
Three scenarios for assessing whether brain aging is a progressive phenomenon. In each case a machine is trained on a young and aged cohort and asked to classify CPs of subjects with ages that it has not previously encountered. For each scenario an illustration contains possible hypotheses (H) for the trajectory of the predicted brain ages in the respective interval. A) In interval 1 it is expected that the percentage of brains classified as aged follow a monotonically increasing trajectory among the chronological age range with the y-intercept being less than 50%. B) The study of interval 2 investigates whether the CP of young brains provide information about the CP of aged brains. C) Interval 3 is intended to investigate whether CP differences among aged brains can be used to infer aging dynamics of younger brains.

To measure FC, PCC values were evaluated between the time courses for all pairs of ROIs of a participant. Each recording consisted of signals collected for 42, 140, and 808 ROIs for subjects in the 1000FCP, SRPBS, and camCAN datasets, respectively. A simple calculation shows that we have 861, 9730, and 326,028 unique PCC values to be used as features. The features from each participant were used either as training or test data in the ML analysis. We form two sets A and B, consisting of a younger age group and an older age group for the scenarios considered in Fig 1. Let N_1_ denote the number of subjects in the young group and N_2_ the number in the aged group. The following steps are taken.

Step 1) Let m = min{*N*_1_, *N*_2_}.

Step 2) Train a machine on the m data points from the smaller set, and m randomly selected points from the larger set. This is done to ensure balance of data for an age range. The feature vectors will consist of the PCC values for a subject. The labels correspond to whether the subject is from the aged or young group.

Step 3) Use the *k* = max{*N*_1_, *N*_2_}−m left-out recordings from the larger set as validation data. The trained machine is to predict whether the k residual recordings are young or aged and an accuracy rate (AR) will be attained.

Step 4) Test on the subjects not included in sets A and B, but rather in interval i: i = 1, 2, or 3 for the Fig 1 scenario that is being considered.

**Fig 2:**
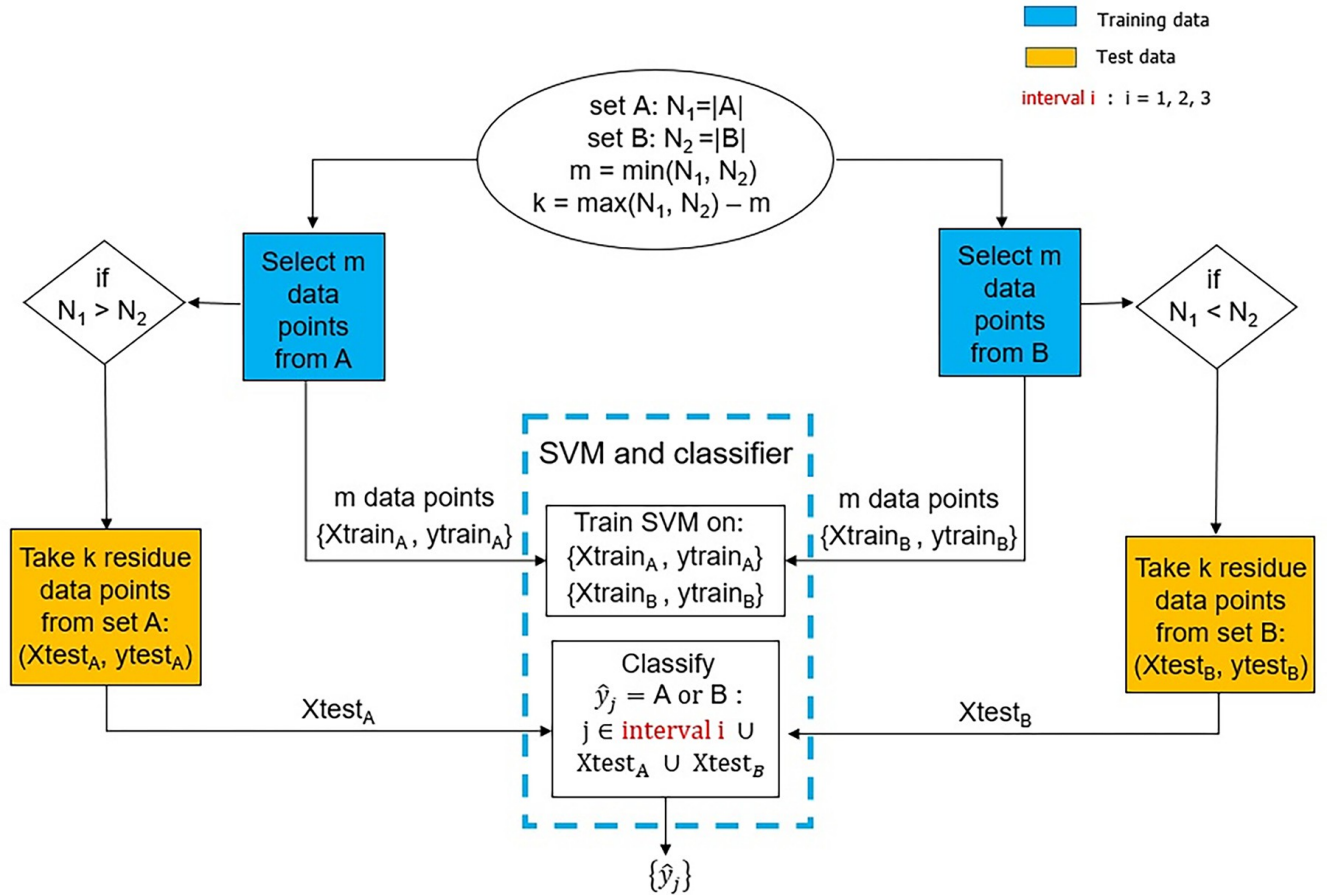
An ML pipeline to evaluate whether brains age in a monotonic fashion. The sets A and B refer to the young and aged groups of the different scenarios that are considered when testing on subjects of interval 1, 2, and 3. An SVM is trained on 2m data points, asked to classify k residue data points from either the aged or young group, and classify the age phenotype of the subjects in interval 1, 2, or 3.

The above steps are depicted in the pipeline of Fig 2 where the schematic is iterated once with the performance on the k residue data points from the larger set viewed as validation accuracy. The trained machine is then presented with the left-out data from the new category. The ML technique used was a support vector machine (SVM) with a linear kernel and a binary classifier. The techniques were implemented in R via the packages e1071 and kernlab.

### Methodology to assess monotonicity of brain differences with age

It is natural to assume that a brain will possess characteristics that are more similar to brains close to its age than those of increasingly disparate age groups. A test of this notion is to present a trained machine with recordings across a spectrum of ages that it has not previously seen. The three scenarios in Fig 1 are considered with the ML pipeline in Fig 2. This will encompass training a machine on connectivity patterns (CPs) from an equal number of subjects from a young and an aged cohort in each scenario. The trained machines’ decisions will scrutinize whether brains–whose age the machine has not previously encountered–have a CP that is more similar to an older or a younger cohort. The cartoons accompanying each of the scenarios in Fig 1 denote possible trajectories that we hypothesize for the left-out recordings that will be classified by the respective machine. Interval 1 is the most conspicuous since it is expected that as chronological age increases, brains exhibit properties that become increasingly similar to an extrema group of aged brains in a monotonic fashion. It is also expected that the youngest brains in the interval exhibit CPs that are close to the extrema of young subjects (i.e., an intercept < 50%). The described relation is referred to as hypothesis 1 (H1) in Fig 1A.

The prospective hypotheses are less conspicuous for interval 2. It is conceivable that older subjects exhibit CPs that follow an increasing trajectory away from younger brains that have been bifurcated into a younger (i.e., aged) and youngest (i.e., young) groups. This possibility is reflected by the two increasing lines labeled H1 and H2 in Fig 1B. The difference in the intercept of the two lines reflects the notion that the youngest brains in interval 2 can be classified as being closer in CP to brains in the aged group that are the most proximal to them in years (H1), or alternatively as relatively young (H2) if the CPs in the unseen data are classified on a relative rather than absolute basis. Lastly, it is conceivable that the recordings exhibit CPs that do not deviate very much from the aged (yet relatively younger) brains in which case a horizontal line with an intercept greater than 50% would be noted (H3). The investigation of interval 2 probes at whether CPs of young brains provide information on the dynamics noted in older subjects.

Interval 3 in Fig 1C represents a somewhat inverted version of the study for interval 2. It investigates whether older brains provide insight into the connectivity changes that occur among younger brains. A hypothesis shown by the strictly increasing line (H1) entails young brains exhibiting properties reminiscent of the youngest brains in an otherwise aged cohort, and the CP of the young brains gradually resembling that of the most aged brains as chronological age is increased. It is also conceivable that the gradual resemblance to the most aged brains does not occur, but rather the trained machine associates the brains in interval 3 as resembling the young cohort as the chronological age of the subjects approaches that of the subjects in the more proximal young cohort. This scenario is depicted via the inverted U-shaped trajectory (H2). Lastly, it may be that the CPs of subjects remain similar to the younger group as shown by the trajectory of a horizontal line with an intercept below 50% (H3). The investigation of interval 3 probes at whether aged brains provide information on the dynamics of connectivity earlier in life.

### Quantification of FC changes with age and sex

The pairwise PCC values were used as the features for the ML analysis and to assess the connectivity among brain regions. For each subject the number of connections that exceeded the threshold |ρ = 0.6| were totaled and referred to as the FC for the subject. The value of 0.6 has been motivated in works such as [19, 25]. For completeness, thresholds of |ρ = 0.45| and |ρ = 0.7| are also considered in the analysis to assess the robustness of the findings. The evaluation with different FC thresholds is to account for the possibility of low SNR in the recordings. Typically, signals in a stable or high SNR regime would provide similar trends when a correlation threshold is uniformly adjusted. Importantly, we consider the positive and negative (i.e. anticorrelation) CC values separately when assessing the change in connectivity with aging for the three datasets. In testing for the statistical significance of an effect, the youngest age group is taken as a reference and a two-sample t-test is conducted among the positive, negative, and total connections in the subjects. A p-value of 0.05 is used as the threshold for statistical significance when evaluating the change in FC between the youngest age group and the older subjects. The change is assessed separately among male and female subjects with the objective of determining whether age-modulated FC is sex specific. As done with the entire population, the youngest age group is considered the reference for assessing FC changes, and a two-sample t-test is used to test for statistical significance (i.e. p < 0.05). The standard deviations (s.d.) of the number of functional connections are also computed to study the inter-subject variability in connectivity during aging.

### Consideration of recording center and subject arousal effects

The 1000FCP dataset consists of recordings from 17 centers, and thus warrants scrutiny of potential site effects. The NKI-Rockland (NKI-RS) center contains approximately 34% of the subjects in the 1000FCP. This number is significant when considering the next largest participant pool (i.e. Beijing) contributes approximately 13%. NKI-RS also contains the largest spread of subjects’ chronological ages (Table 1). The monotonicity and change in FC analysis shall be repeated with only subjects from the NKI-RS center. A comparison of the findings for 1000FCP and its constituent NKI-RS will show whether the same trends are noted for both cases or if site effects may be affecting the findings. Furthermore, unlike SRPBS and camCAN, the 1000FCP contains a mixture of recordings where subjects had their eyes open and closed (Table 2). A difference in arousal level can affect FC strength and bias connectivity patterns. The monotonicity and change in FC analyses are conducted separately on 1000FCP recordings where subjects had their eyes open (N = 543). The reason for not additionally conducting an analysis on subjects with eyes closed is because of their relatively low number (N = 197), and lack of representation across the spectrum of ages in the 1000FCP dataset (Table 2).

## Results

### Monotonic changes in functional connectivity occur over an intermediate age interval

**Fig 3:**
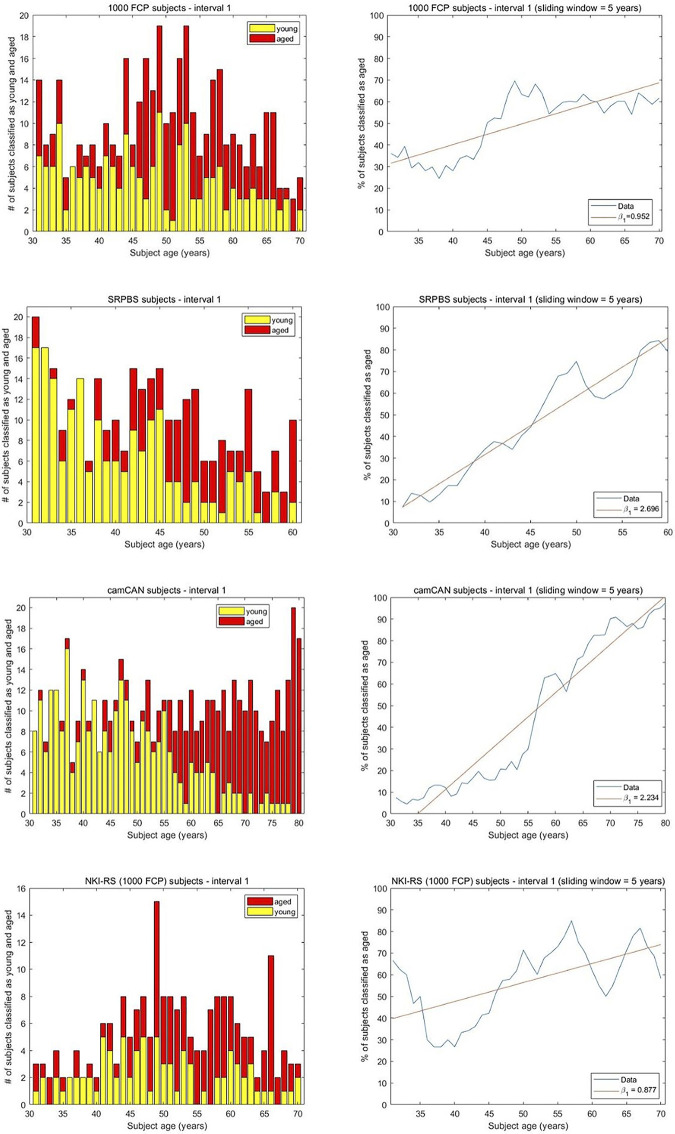
The results of the brain age monotonicity analysis for interval 1. The histograms show the number of subjects classified as young and aged at every considered age. A 5-year sliding window was applied and a linear regression was fit to the results with intercept β_0_ and slope β_1_. A machine was trained on the youngest and oldest groups and presented with test data of intermediate-aged subjects. For 1000FCP we have β_0_ = 31.58, β_1_ = 0.952. The Spearman correlation of 0.7 computed among the percentages of subjects classified as aged and the ages indicates a moderate degree of monotonicity between the two quantities. With SRPBS, β_0_ = 7.285, β_1_ = 2.696, and a Spearman correlation of 0.948 demonstrates a high degree of monotonicity between the two quantities. A consideration of camCAN leads to β_0_ = 0 and β_1_ = 2.234. The Spearman correlation of 0.986 signifies monotonicity between the two quantities. For NKI-RS subjects we note β_0_ = 39.74, β_1_ = 0.877, and the Spearman correlation of 0.608 reflects monotonicity.

In considering the 1000FCP data, the scenario in Fig 1A encompassed a machine trained on features from 78 brains, 39 that comprise the 71+ age group and 39 of which have been randomly selected from the 21–30 group. The remaining 809 brains shall be considered as test data. Naturally, it is expected that the 419 remaining recordings from the 21–30 age group be classified as young. In evaluating the accuracy on the test data, excellent performance is seen for the left-out young brains in the 21–30 age group via an AR of 0.84 across 419 subjects. The accuracy vindicated the machine’s integrity to continue the monotonicity study and evaluate decisions on recordings in interval 1. The decisions made by the machine on the 390 brains of ages 31–70 years are shown in Fig 3. We have considered the number of brains at each age that were classified as old to arrive at percentages for the ages encompassed in interval 1. The bar plot illustrates the number of brains at an age that have been classified as young or aged. The accompanying line plot represents the percentage of subjects that were classified as aged after the application of a 5-year sliding window for smoothing. Although the increase is not monotonic, an increasing trend is noted in the percentage. The absence of complete monotonicity is due to cross-subject variability as variance inherently exists among all facets of recordings from different people. Furthermore, there is the limitation that the number of samples at each age in Fig 3 was not very large (min sample size = 3, max sample size = 19). Such confounding factors are natural and expected to provide uncertainty to the findings. This is noted by the moderate Spearman correlation value of 0.7 between the subjects’ ages and the percentage of subjects classified as aged. To test for a trend, a linear regression with intercept β_0_ and slope β_1_ was fit to the results. As previously mentioned, a positive trend and a low intercept are expected for the brains in interval 1. The intercept β_0_ = 31.58 is rather low indicating that the classification of the youngest brains in interval 1 are indeed skewed towards the young group. A slope of β_1_ = 0.952 demonstrates a positive association between the increase in age and CPs resembling the older phenotype. The results justify H1 as holding for interval 1 of Fig 1A.

For the SRPBS subjects, a machine is trained on 72 subjects randomly selected from the 18–30 age group and the 72 in the 61+ group. The test set consists of 565 subjects, 255 of which are residual from the 18–30 year old (y.o.) range. The machine classified 247 of the residual recordings as young leading to AR = 0.968 and vindicating the machine efficacy. The histograms in Fig 3 illustrate the number of subjects assigned as young and aged at every considered age. To test for a trend, a linear regression was applied to the results in interval 1. The low intercept (β_0_ = 7.285) and the positive slope (β_1_ = 2.696) were expected while a Spearman correlation of 0.948 indicates a high degree of monotonicity in the brains classified as having an aged profile with increasing biological age. The hypothesis H1 in Fig 1A is validated. Similarly, for the camCAN dataset a machine was trained on 45 randomly selected subjects from the 18–30 age group and the 45 subjects 81+ y.o. Of the 34 remaining subjects in the younger group 33 were classified as young leading to AR = 0.9705. A linear fit to the percentage of subjects classified as aged in Fig 3 yields the intercept β_0_ = 0 and slope β_1_ = 2.234. The Spearman correlation of 0.986 computed among the percentages of subjects classified as aged and their chronological ages indicates monotonicity between the two quantities. The analysis supports H1 as the brain aging dynamics of intermediate aged subjects when extrema groups are used as the references. The NKI-RS analysis entailed a machine trained on features from 32 recordings of subjects in the 71+ age group and 32 randomly selected from the 21–30 age group. The remaining 33 recordings from the young group (21–30 y.o.) were classified by the trained machine at an AR of 0.696. The decisions on the residual 210 brains spanning 31–70 years are shown in Fig 3. An increasing trend (β_1_ = 0.877), relatively low intercept (β_0_ = 39.74), and a formidable Spearman correlation value of 0.608 are noted. The results also support hypothesis H1 as an explanation for brain aging dynamics in interval 1.

### Young brain functional connectivity is not indicative of alterations in older subjects

**Fig 4:**
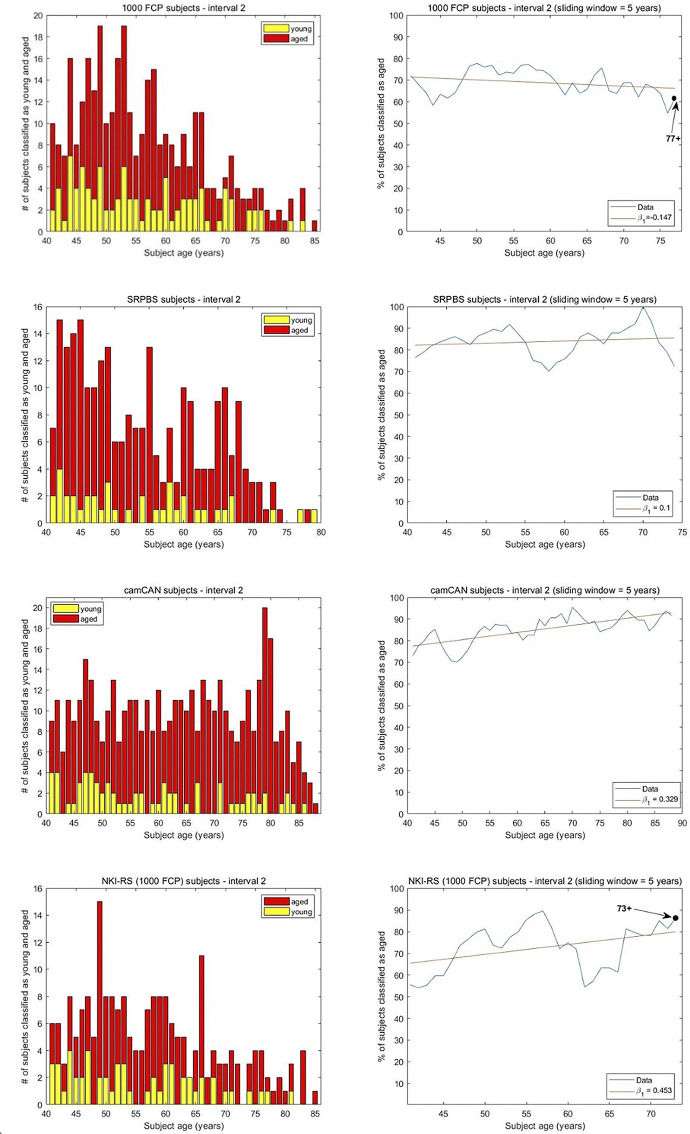
The results of the brain age monotonicity analysis for interval 2. The histograms show the number of subjects classified as young and aged at every considered age. A 5-year sliding window was applied and a linear regression was fit to the results with intercept β_0_ and slope β_1_. A machine was trained on the two youngest groups of subjects and then asked to classify older recordings. For 1000FCP we attain β_0_ = 71.4 and β_1_ = -0.147 indicating little change in the classification of increasingly older brains as aged. Furthermore, a Spearman correlation of -0.243 reflects a non-monotonic relationship. With the SRPBS subjects β_0_ = 82.159 and β_1_ = 0.1 suggest little change in the classification of increasingly older brains as aged. The Spearman correlation of 0.149 reflects a non-monotonic relationship among the ages and the percentage classified as aged. For camCAN we attain β_0_ = 77.48, β_1_ = 0.329 demonstrating little change in the classification of increasingly older brains as aged. The Spearman correlation of 0.74 reflects a moderately monotonic relationship among the subjects’ ages and the percentage classified as aged. The consideration of NKI-RS subjects of interval 2 leads to β_0_ = 65.48, β_1_ = 0.453, and a Spearman correlation of 0.417. This indicates an increasing relationship but little monotonicity.

Considering the 1000FCP subjects, the ML pipeline is applied with the partition in Fig 1B to probe whether there is a monotonic relationship. A machine is trained on 170 brains, 85 of which have been randomly selected from the 21–30 age group, and 85 that comprise the 31–40 age group. The remaining 717 subjects in the dataset are used to investigate whether brains are perceived as young or aged by the machine. The 373 remaining brains from the 21–30 age group are expected to be classified as young, however, the decisions are not obvious for the 344 brains that constitute interval 2. The AR noted is 0.646 for the held-out brains in the young group. It is expected that the AR levels for Fig 1B be lower than those reported for Fig 1A–this is attributed to the difficulty in distinguishing among brains that are closer in age. We consider the decisions made by the trained machine on the 344 elder brains (41–71+ y.o.) of interval 2. In Fig 4 the percentage of brains classified as aged does not show a monotonically increasing relationship (Spearman correlation = -0.243). The point 77+ on the abscissa is considered as the final age because of the relatively few samples available above this age–the point is comprised of 14 subjects ranging from 77 to 85 y.o. When fit with a linear regression, the intercept β_0_ = 71.4 is rather high indicating that the youngest brains in interval 2 appear more similar to the proximal brains in the aged group rather than the relatively young group that was used during training. The slope of β_1_ = -0.147 does not support an increasing trend for the 41+ y.o. subjects of interval 2. The Spearman correlation of -0.243 between chronological age and the percentage of brains classified as aged indicates that the change in CP dynamics of the younger brains is not conserved among the progressively older brains. We conclude that hypothesis H3 in Fig 1B is the most representative trajectory for interval 2.

Applying the analysis to the SRPBS dataset involved a machine trained on 126 subjects randomly selected from the 18–30 age group and 126 comprising the 31–40 group. The test set consisted of the remaining 457 subjects. Of the 201 residual subjects from the 18–30 y.o. group, 146 were classified as young leading to AR = 0.726. In interval 2 we attain intercept and slope values of β_0_ = 82.159, β_1_ = 0.1 indicating little change in the appearance of increasingly older brains from the more proximal aged group. A Spearman correlation of 0.149 in Fig 4 denotes negligible monotonicity in the percentage of subjects classified as aged with an increasing number of years. The findings support hypothesis H3 that studying the aging trajectory in younger brains (i.e. 18–40 y.o.) provides little information about their aging trajectories in later years. Analysis with the camCAN subjects considered the training data comprised of 79 subjects from the 18–30 as well as the 31–40 age groups, and testing done on the 493 residual subjects. The 26 subjects from the older group were classified as aged at an AR of 0.653 by the trained machine. After computing the percentages of 468 subjects deemed as old at each age in the 41+ range, the linear fit provided β_0_ = 77.48 and β_1_ = 0.329. This indicates little increase in the classification of the older subjects with the increase in their chronological ages. The Spearman correlation of 0.74 between the percentage of brains classified as aged and the chronological age reflects a moderately monotonic relationship. The findings signify H1 as the most expressive of the hypotheses for the trajectory in interval 2. NKI-RS subjects in the age group 21–30 and 31–40 were used to train an SVM for the interval 2 analysis. Following the selection of 27 random recordings from the younger group, the 38 left out recordings were classified at a below-chance level of AR = 0.473. By computing the percentages of the 215 subjects that were 41+ y.o. and classified as aged, a linear fit provided β_0_ = 65.48 and β_1_ = 0.453 (Fig 4). While the slope indicates an increasing trend, a Spearman correlation of 0.417 between the percentage of brains classified as aged and the subjects’ ages in interval 2 reflects the absence of a monotonic relationship. The analysis signifies H3 as the most representative hypothesis for the NKI-RS trajectory in interval 2.

### Connectivity patterns of older subjects reflects aging dynamics in young brains

**Fig 5:**
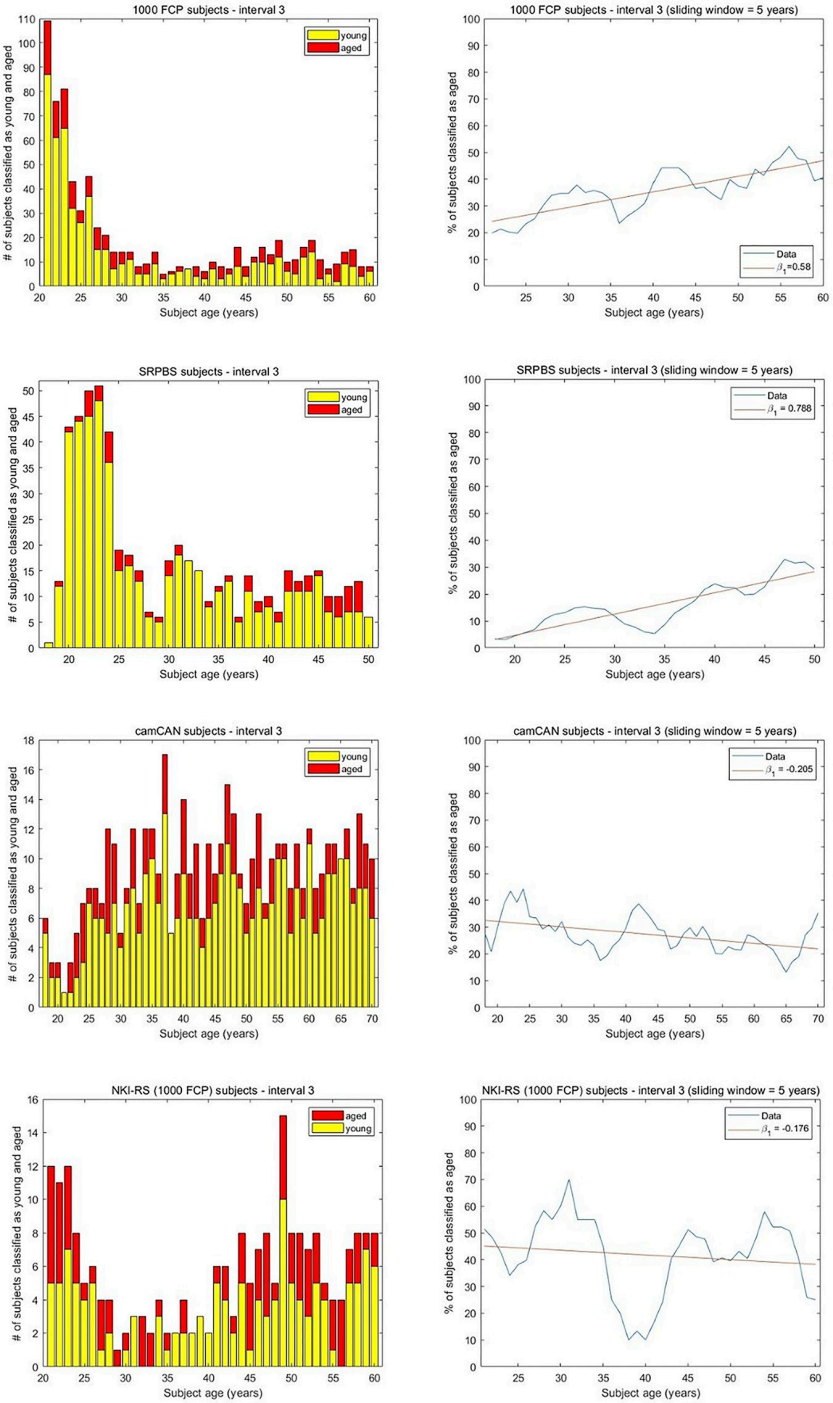
The results of the brain age monotonicity analysis for interval 3. The histograms show the number of subjects classified as young and aged at every considered age. A 5-year sliding window was applied and a linear regression was fit to the results with intercept β_0_ and slope β_1_. A machine was trained on the two oldest age groups and received younger subject recordings as test data. With the 1000FCP dataset, the values β_0_ = 24.2, β_1_ = 0.58, and the Spearman correlation of 0.795 indicate a gradual increase and moderate monotonicity. For SRPBS, β_0_ = 3.11, β_1_ = 0.788, and the Spearman correlation of 0.853 demonstrates monotonicity in the number of subjects classified as aged with increasing age. With camCAN, the values β_0_ = 32.52, β_1_ = -0.205, and the Spearman correlation of -0.448 indicate a gradual decrease and small degree of monotonicity. The NKI-RS subjects of interval 3 provide β_0_ = 45.12 and β_1_ = -0.176 to reflect a gradually decreasing relationship. The Spearman correlation of -0.161 indicates an absence of monotonicity.

Through the scenario depicted in Fig 1C we explore if older brains exhibit CPs that can be used to differentiate among younger brains, and whether the differentiation occurs in a monotonic fashion with age. We hypothesize the trained machine to provide a low intercept when investigating a trend in the percentage of brains classified as aged. However, it is not obvious if a monotonic increase or a positive association will be seen. For the 1000FCP dataset an SVM is trained on 78 brains; 39 randomly selected from the 61–70 age group and 39 from the 71+ age group. The residual subjects from the 61–70 age group are expected to be classified as young, and an AR of 0.714 was noted on the 28 held-out brains from this group. The decisions made by the trained machine on the 781 younger brains (ages 21–60 years) are shown in Fig 5. The intercept of β_0_ = 24.2 is somewhat high since the ideal intercept would be near zero. This demonstrates that the relatively young brains that were used to train the machine are not very representative of the FC changes in the youngest subjects of the study. The slope in Fig 5 is β_1_ = 0.58 indicating a marginal but increasing trend in the percentage of brains classified as aged across interval 3. A Spearman correlation of 0.795 is noted among the subjects’ ages and the percentage of brains classified as aged. We conclude that H1 is the best hypothesis for interval 3. This indicates that the difference in CP among aged brains is reflective of FC dynamics earlier in life.

Applying the ML pipeline to the SRPBS dataset, a machine is trained on 69 subjects in the 51–60 age group and 69 subjects randomly selected from the 61+ group. The testing was performed on the remaining 571 subjects. Of the 3 remaining subjects from the 61+ y.o. group, 2 were correctly classified as aged (AR = 0.667). By applying the ML pipeline of Fig 2 to this scenario, we attain the results in Fig 5 for interval 3. A linear fit provides a low intercept (β_0_ = 3.11) and a positive slope of β_1_ = 0.788. The Spearman correlation of 0.853 signifies that differences among aged brains (i.e. 61+ y.o.) provide a high degree of information about the aging trajectory of younger brains. The quantified results indicate that hypothesis H1 is the most explanatory of the three hypotheses for interval 3. The study with the camCAN subjects involved training on 45 young (71–80 y.o.) and 45 aged (81+ y.o.) subjects and subsequently testing on the 561 residual recordings. The 72 residue subjects in the 71–80 group were classified accurately as young via AR = 0.666. For the remaining 489 recordings that comprise interval 3, the linear fit provides β_0_ = 32.52 and β_1_ = -0.205. The Spearman correlation of -0.448 reflects a small degree of monotonicity in the number of subjects classified as aged when the reference was taken to be the aging dynamics of older brains. The scenario H3 is the endorsed hypothesis as the properties of the two chronologically oldest groups do not markedly differentiate between the ages of the recordings from the younger subjects. The ML analysis for the NKI-RS subjects involved training on 32 young (61–70 y.o.) and 32 aged (71+ y.o.) recordings. The 13 residual subjects in the 61–70 group were classified as young via AR = 0.692. The decisions made by the machine on the 230 younger subjects comprising interval 3 yield β_0_ = 45.12 and β_1_ = -0.176 when fit with a linear model (Fig 5). A Spearman correlation of -0.161 does not reflect monotonicity and H3 is recognized as the most supported hypothesis.

### Functional connectivity changes are categorical with aging

**Fig 6:**
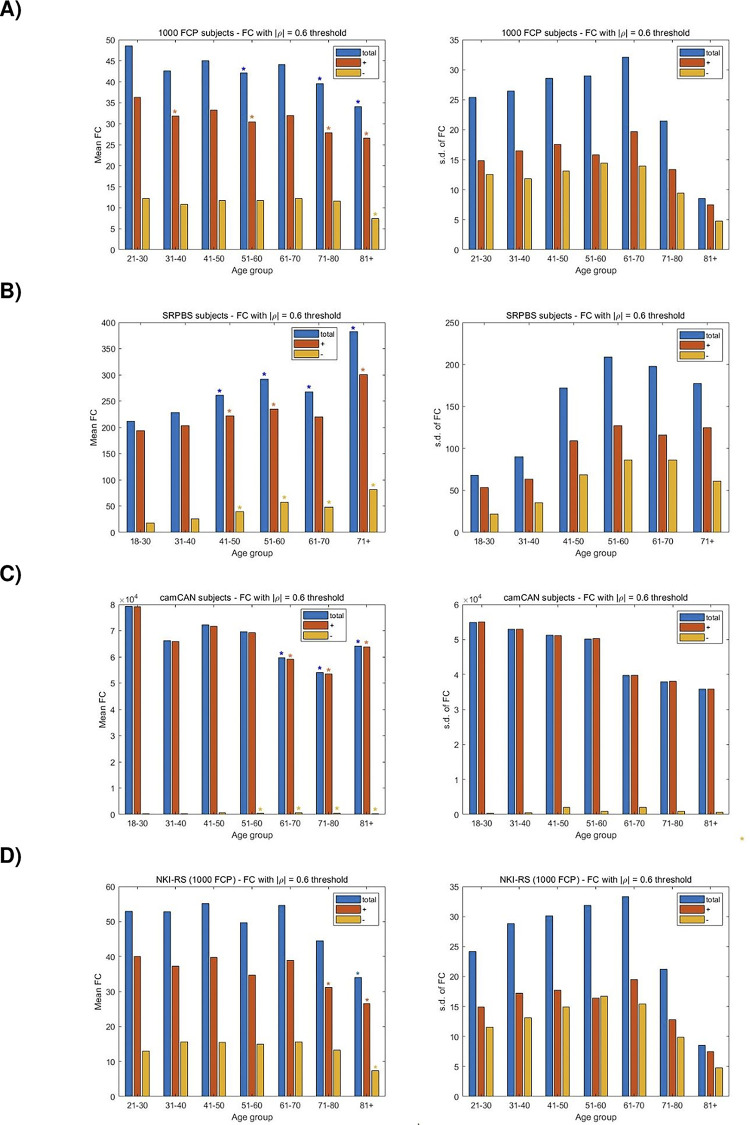
A study of the change in FC for subjects across decades from the A) 1000FCP, B) SRPBS, C) camCAN, and D) the NKI-RS recording center. The mean FC was computed by averaging across subjects the number of times that the Pearson CCs from the connectivity matrix exceeded a threshold of |ρ| = 0.6. The bifurcation of the FC in positive and negative (i.e. anticorrelations) directions is also shown. Two-sample t-tests are used to assess the significance of the change in FC with the youngest age group taken as the reference. A p-value of 0.05 is the threshold for statistical significance in the pairwise comparisons between positive, negative, and total FC values. The s.d. of the number of functional connections is illustrated to study the inter-subject variability across the age spectrum.

In light of the ML analysis providing predictions from measures of FC, we pursue a quantitative evaluation of the FC and how it varies across the age spectrum. For the 1000FCP dataset, Fig 6A shows the mean and standard deviation (s.d.) of the FC for subjects across the seven decades. A significant decrease in mean FC in noted among the 51–60, 71–80, and 81+ groups (p < 0.05). The positive FC values that showed a significant decrease coincided with the total FC except for the 31–40 age group. Although less apparent, the presence of anticorrelations was not negligible, and the decrease in the negative FC values was significant for the 81+ group. The alteration in FC with aging was also prevalent for the SRPBS subjects, however the direction of the change was different as there is an increase in FC with age (Fig 6B). The increase accelerated among the 51–60 and 71+ groups and, aside for the positive correlations in the 61–70 y.o. group, is seen for positive, negative, and total connectivity measures. The change in the number of negative connections is not insignificant and follows an increasing trend that coincides with the conventionally considered (i.e. positive) correlations. The camCAN subjects show a decrease in FC that is steady but significant with increasing age (Fig 6C). Interestingly, while being negligible in value in comparison to the number of positive correlations, the anticorrelations show an opposite trajectory by increasing with aging. For the NKI-RS dataset, Fig 6D shows the mean of the FC for subjects. A significant decrease in mean FC is noted among the 81+ y.o. group and for the positive FC values of the 71–80 y.o. group (p < 0.05).

We examine the inter-subject variability in FC with aging. In the case of 1000FCP, there is an increase in variability among middle age to elder (61–70) subjects, and a reduction in FC variability for the two oldest decades (Fig 6A). It is intriguing that the increase in FC consistency for the oldest subjects is precipitous and present among the positive and negative correlations. Similarly, the peak inter-subject FC variability occurs in middle age for the SRPBS subjects (51–60 y.o.) with decreasing variance for older and younger age groups (Fig 6B). The youngest subjects (18–30 y.o.) exhibit the most consistency. The variance in the inter-subject FC for the camCAN subjects shows a steady decrease along the decades. There is a bimodal division as the older subjects (61+ y.o.) have a more consistent CP than the younger subjects (Fig 6C). It is interesting that the datasets provide very different results on the variability in inter-subject FC across the broad age spectrum. While the 1000FCP and camCAN subjects show the least variability in the oldest age groups, the SRPBS subjects show the lowest variability for the youngest subjects. Furthermore, the 1000FCP and SRPBS subjects show the highest variability for middle-age (61–70 and 51–60, respectively), while the youngest decade (18–30 y.o.) camCAN subjects exhibited the largest inter-subject FC variability. In the case of the NKI-RS subjects there is an increase in variability among middle age to elder (61–70 y.o.) subjects, and a reduction in FC variability for the two oldest decades (Fig 6D). The trend is very similar to the aggregate 1000FCP scenario since the increase in FC consistency is precipitous for the oldest subjects and exists for positive and negative correlations. In summary, the change in the total FC is categorical with aging across the populations of subjects. The inter-subject variance, however, follows an inverted U-shaped trajectory in two datasets, and a bimodal trajectory showing a decrease in variability with aging in the third case.

### Brain aging dynamics are similar among sexes, but accentuated in males

**Fig 7:**
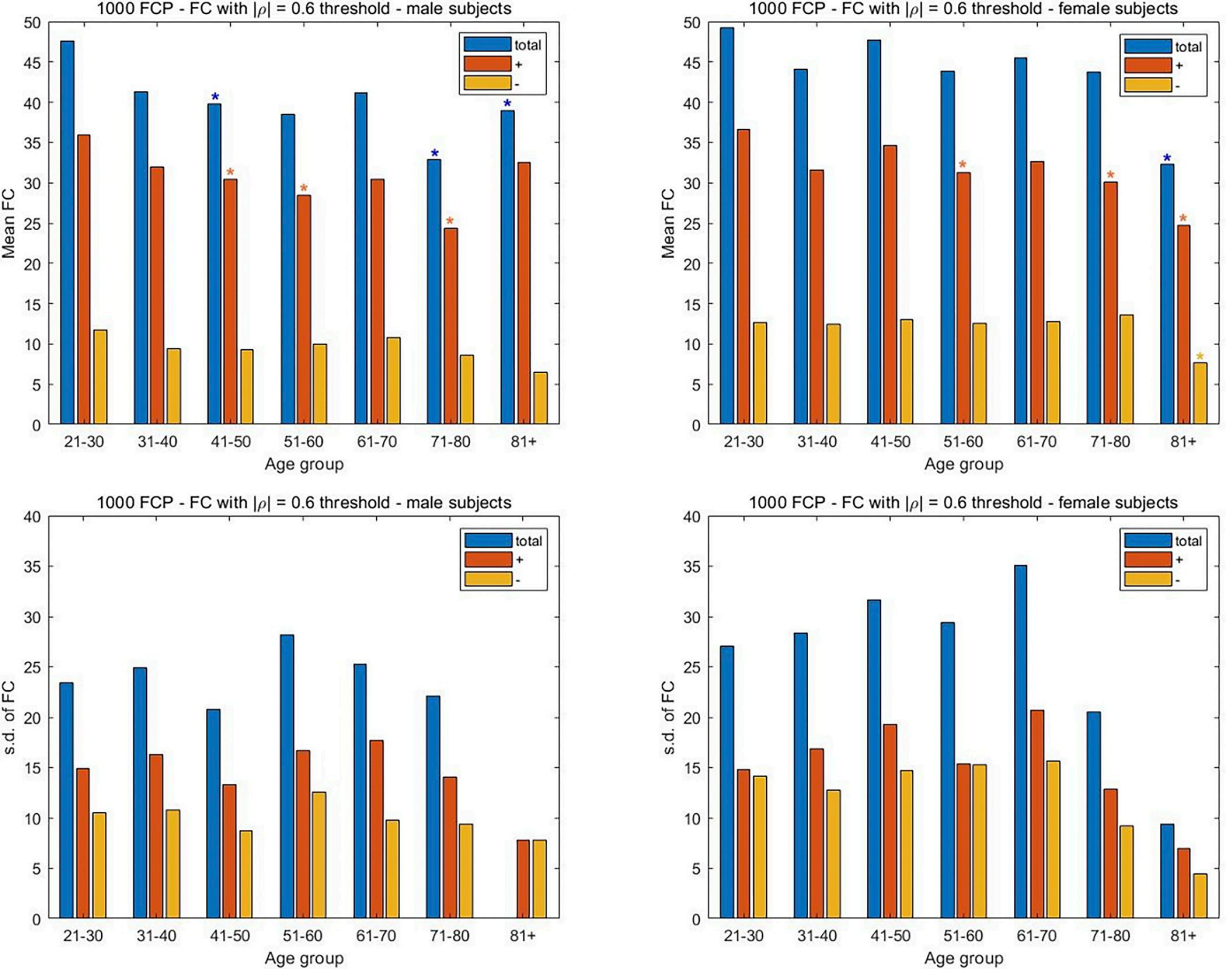
An assessment of the differences in FC between male and female subjects in the three datasets. The mean and s.d. of the number of connections are plotted separately for the male and female subjects from the 1000FCP dataset. The mean FC was computed by averaging across subjects the number of times the Pearson CCs from the connectivity matrix exceeded a threshold of |ρ| = 0.6. The division of the FC in positive and negative (i.e. anticorrelation) directions is also shown. Two-sample t-tests are used to assess the significance of the change in FC with the youngest age group taken as the reference. A p-value of 0.05 is the threshold for statistical significance in the pairwise comparisons between positive, negative, and total FC values. The s.d. of the number of FCs is shown to study the inter-subject variability for each sex and age group.

**Fig 8:**
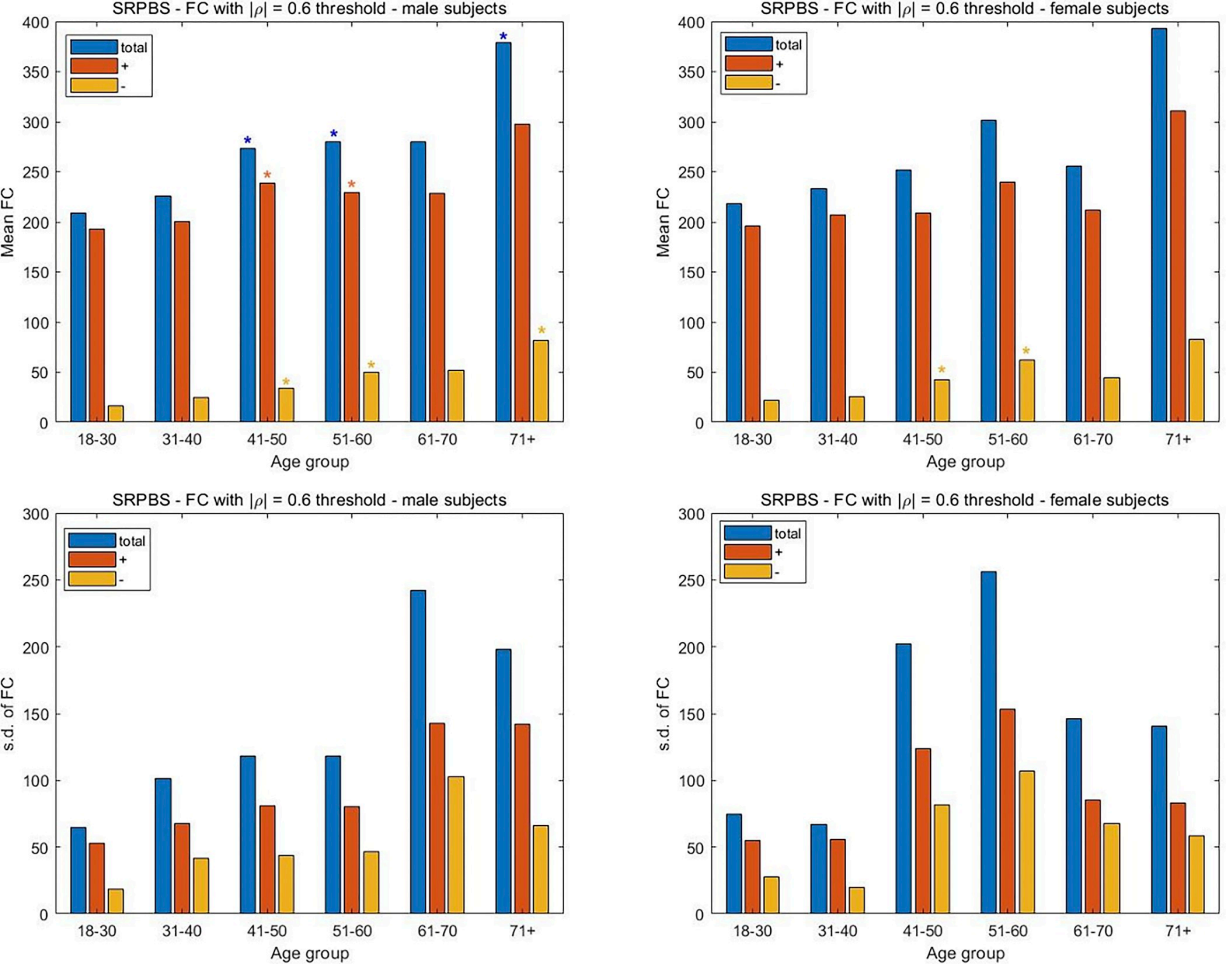
Assessment of the differences in FC between male and female subjects in the three datasets. The mean and s.d. of the number of connections are plotted separately for the male and female subjects from the SRPBS dataset. The mean FC was computed by averaging across subjects the number of times the Pearson CCs from the connectivity matrix exceeded a threshold of |ρ| = 0.6. The division of the FC in positive and negative (i.e. anticorrelation) directions is also shown. Two-sample t-tests are used to assess the significance of the change in FC with the youngest age group taken as the reference. A p-value of 0.05 is the threshold for statistical significance in the pairwise comparisons between positive, negative, and total FC values. The s.d. of the number of FCs is shown to study the inter-subject variability for each sex and age group.

**Fig 9:**
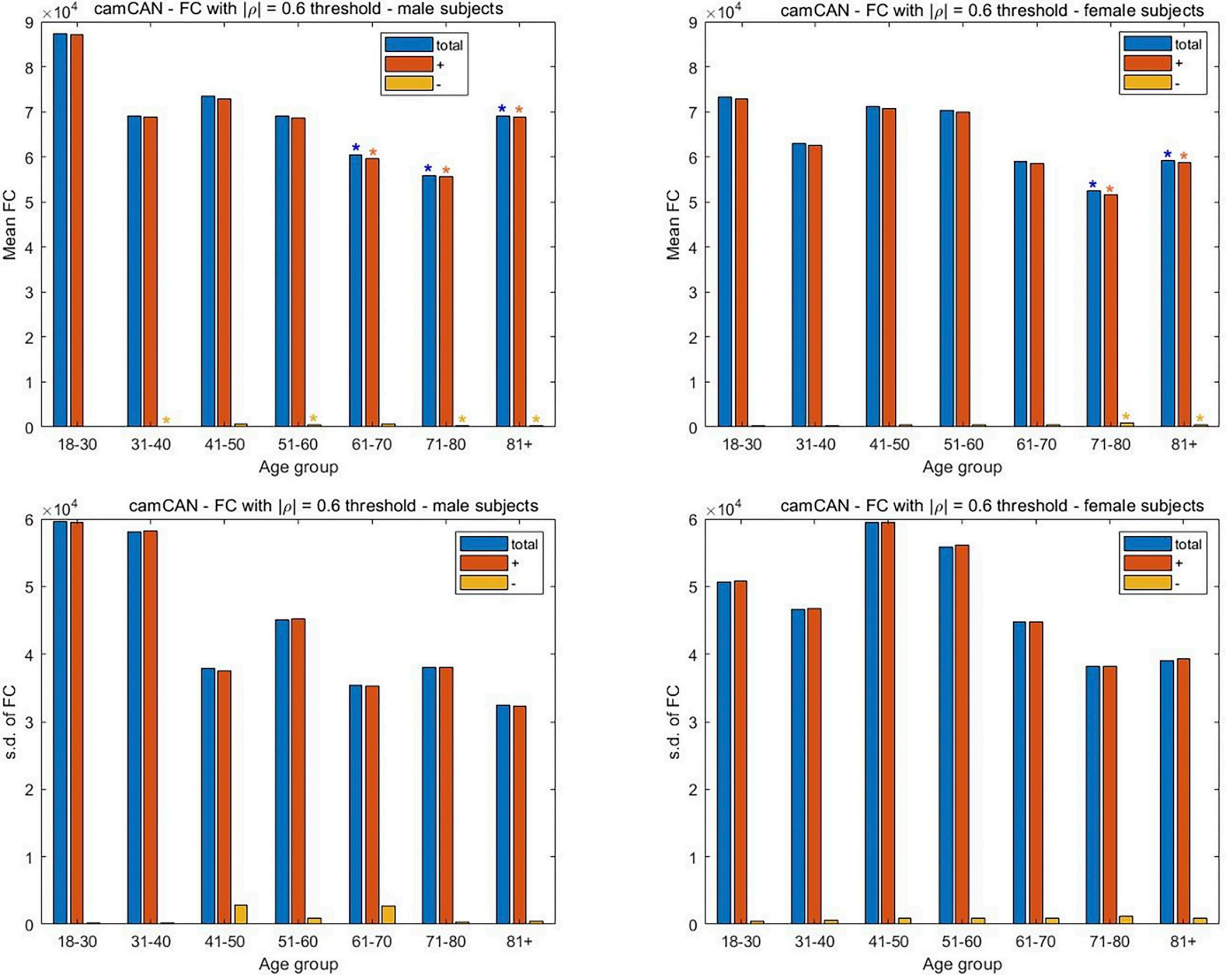
Assessment of the differences in FC between male and female subjects in the three datasets. The mean and s.d. of the number of connections are plotted separately for the male and female subjects from camCAN. The mean FC was computed by averaging across subjects the number of times the Pearson CCs from the connectivity matrix exceeded a threshold of |ρ| = 0.6. The division of the FC in positive and negative (i.e. anticorrelation) directions is also shown. Two-sample t-tests are used to assess the significance of the change in FC with the youngest age group taken as the reference. A p-value of 0.05 is the threshold for statistical significance in the pairwise comparisons between positive, negative, and total FC values. The s.d. of the number of FCs is shown to study the inter-subject variability for each sex and age group.

**Fig 10:**
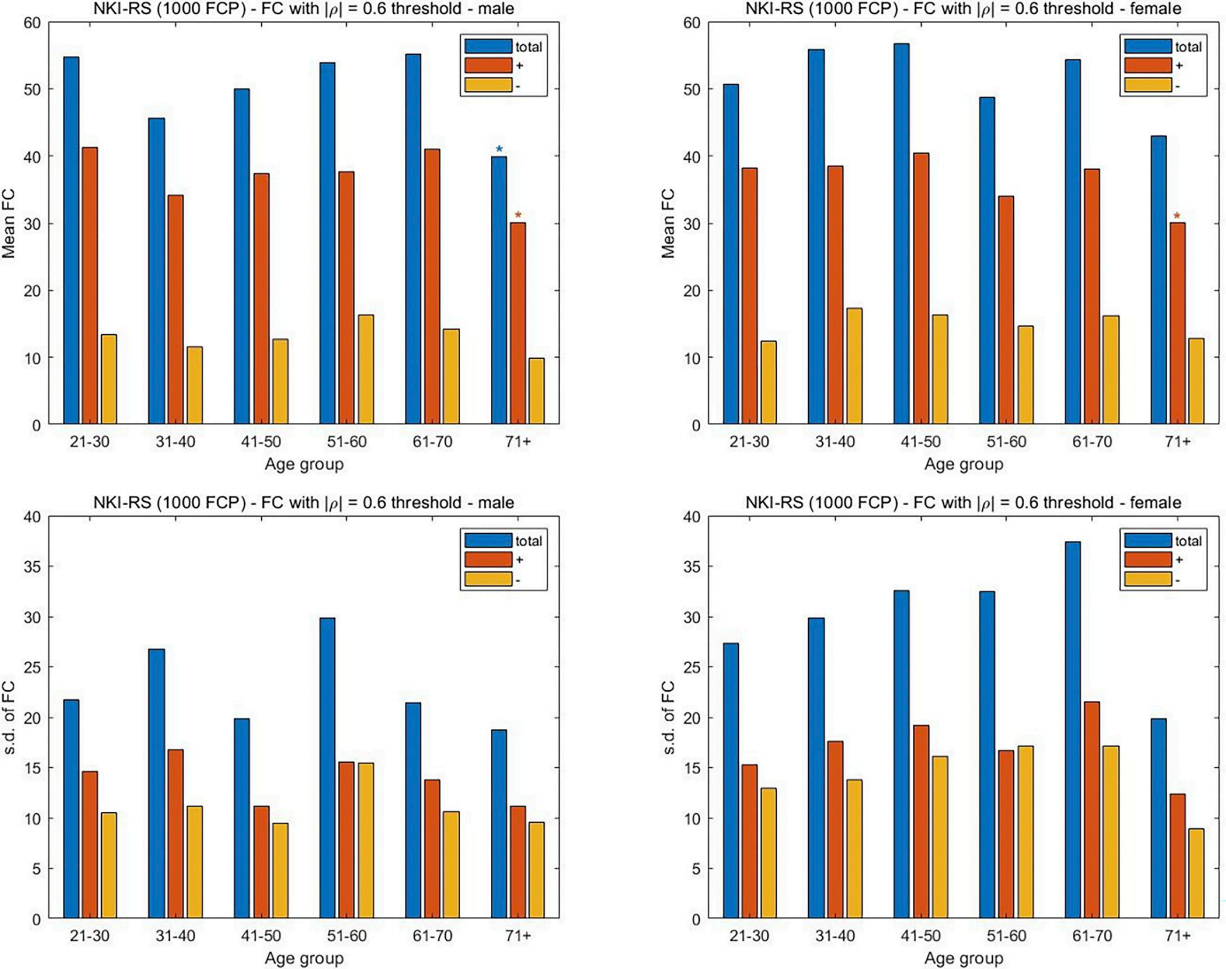
Assessment of the differences in FC between male and female subjects in the three datasets. The mean and s.d. of the number of connections are plotted separately for the male and female subjects from the NKI-RS recording center. The mean FC was computed by averaging across subjects the number of times the Pearson CCs from the connectivity matrix exceeded a threshold of |ρ| = 0.6. The division of the FC in positive and negative (i.e. anticorrelation) directions is also shown. Two-sample t-tests are used to assess the significance of the change in FC with the youngest age group taken as the reference. A p-value of 0.05 is the threshold for statistical significance in the pairwise comparisons between positive, negative, and total FC values. The s.d. of the number of FCs is shown to study the inter-subject variability for each sex and age group.

In considering the 1000FCP subjects, the results in Fig 7 indicate that a decrease in FC with increasing age is more pronounced in males. Specifically, the two middle-age groups of males (41–50, 51–60) exhibited a significant decrease in their positive FC measures (p < 0.05) while only the 51–60 y.o. group of females showed a similar decline. The significant decrease in FC is noted in the total FC of males for the two oldest groups. A similar decrease is noted in females of 81 years and older. Interestingly, the oldest females witness a greater reduction in FC than the male subjects. For the SRPBS dataset there is an increase in FC with aging for both males and females, although the increase is only significant for the male subjects. Specifically, the increase in all three classes of correlations are significant for the male subjects in the 41–50 and 51–60 y.o. range, while only the negative correlations are significant for the female subjects of the same age groups (Fig 8). Perhaps more importantly, the males show their largest number of negative and total connections in the oldest age group. The subjects in the camCAN cohort exhibit a similar trend with the progressive change in FC being more prominent in males. While a significant decrease in total FC is noted in the 71–80 and 81+ age groups for both sexes, the change starts earlier in males via the 61–70 y.o. group (Fig 9). The earlier alteration to FC in males is also noted among their anticorrelations across the age spectrum. The change in FC for the NKI-RS subjects is similar to that seen for the aggregate 1000FCP dataset (Fig 10). The decrease in connectivity is only significant for the oldest age group of male and female subjects. The change is more pronounced in males because the total and positive FC values are statistically significant, while only the positive FC values are significant for females.

We examine the inter-subject variability in FC with aging separately among the sexes. A rising and precipitous fall in variability is noted among both sexes for the 1000FCP subjects (Fig 7). However, the peak variability occurs at the different junctures of 51–60 for males and 61–70 for females when considering the total FC. A similar inverted U-shape trend is noted with the SRPBS data (Fig 8) but with the peak variance in inter-subject FC at the 61–70 age group in males while the peak variability for females occurred during the 51–60 interval. In Fig 9, the camCAN results for the female subjects showed a similar spike in inter-subject variability during middle age since the 41–50 and 51–60 year-old females had the least consistency. The males show a variation in FC that is incongruous to what we have noted thus far. Namely, they showed the greatest variance in the young (18–30 and 31–40) and a relatively constant variance in the latter decades. The NKI-RS male and female subjects show a rising and precipitous falling in FC variability with aging (Fig 10). The trends are similar to that for the aggregate 1000FCP since the peak variabilities occur at 51–60 for males and 61–70 for females. In both sexes the lowest variability is seen for the oldest age group.

## Discussion

In the course of healthy human aging, one may expect a monotonic relationship with young brains having properties that are increasingly similar to aged brains. The application of ML pipelines can aid in the discovery of fundamental facets of brain aging including the role of sex. While our use of SVM was motivated by prior works [5, 26], the questions and classification-based methodology are novel. The three intervals studied in Fig 1 investigate questions pertaining to the dynamics of brain aging among healthy subjects. A strong degree of monotonicity was noted when considering the two extrema age groups as references (interval 1 of Fig 1A). This was consistent among three datasets that contained subjects of diverse demographics. A discrepancy from a Spearman correlation value of one–i.e., a completely monotonic relationship–points towards several practical considerations. For instance, the study is prone to inter-subject variability since participants of the same age may have very different genetic traits, environmental factors, and life histories while participants in a different age group may be more similar to each other with respect to the aforementioned factors.

The analysis that accommodated a new question of whether the CPs of young brains contain structure that is conserved among progressively older brains (interval 2 of Fig 1B) provides a less categorical answer. While the majority of brains were classified as more similar to the aged group, there was little indication of monotonicity in the classifiers’ decisions across the age spectrum. The difference in CP among younger brains was not indicative of nor resonated to the CP changes of the older brains. Conversely, it is interesting that information can be gained about the connectivity of young brains through the CPs of strictly older brains (i.e. interval 3 of Fig 1C). In two of the datasets a linear fit showed that the CP associated with old subjects (61+ y.o.) are informative of relative changes in much younger subjects. Table 3 contains the prominent hypotheses among the possible brain aging trajectories in each interval for the datasets. The ML parameters as well as the results attained via the post-processing are also listed. To the best of our knowledge, this work presents the first evaluation of monotonicity in brain aging and its quantification via ML. The analysis has gone beyond the paradigm of brain age prediction from fMRI signals or changes in brain connectivity to probe whether differences in CPs of young brains are reflective of the differences in later life. Interestingly, the complementary question of whether the difference in CPs of aged brains are informative of the changes seen by younger subjects provided a different answer. For each of the three intervals in the monotonicity study, a 5-year sliding window was applied for smoothing. This was done prior to fitting a linear regression to the percentage of subjects that were classified as aged across the spectrum. The analysis was also performed without the sliding window (S1 Fig) and we did not witness a change in the conclusions. It is noteworthy that the Spearman CC values were smaller in magnitude without the sliding window. This is expected because not smoothing may obfuscate or hide trends if the data at a single age contains outliers or when fewer, non-representative subjects exist at an age. The interval 1 subjects in Figs 3–5 that deviate from a monotonic relationship deserve further discussion. This is explained by the limitation in the number of subjects as well as the empirical nature of the collected data. Despite a smoothing operation having been applied, the percentage of subjects classified as aged was computed for every year in the interval. This temporal resolution further contributed to specific ages consisting of a fewer number of subjects and the likelihood of a non-representative CP affecting the results at a particular age. Another factor follows from the choice of the monotonicity measure. Although a commonly used metric, the Spearman CC is a measure of linear monotonicity. Conversely, the BOLD signal as well as the aging process are nonlinear processes at both the micro- and macroscale. As increased data, analysis, and results come forth, it will perhaps be possible to thoroughly assess the suitability of nonlinear measures of monotonicity for explaining brain aging dynamics.

**Table 3:** The comparative parameters and results of the monotonicity study conducted with the three datasets. The intercept (β_0_) and slope (β_1_) attained via a linear fit to the percentage of subjects classified as aged in intervals 1, 2, and 3 are included. The Spearman CC and fitted parameters were used to determine the prominent hypothesis among the possible brain aging trajectories.

Interval	Dataset	Size of training set	Size of test set	*β*_0_	*β*_1_	Spearman CC	Prominent hypothesis
1	1000FCP	78	390	31.58	0.952	0.7	H1
1	SRPBS	144	310	7.285	2.696	0.948	H1
1	camCAN	90	528	0	2.234	0.986	H1
1	NKI-RS	64	210	39.74	0.877	0.608	H1
2	1000FCP	170	344	71.4	-0.147	-0.243	H3
2	SRPBS	252	256	82.159	0.1	0.149	H3
2	camCAN	158	468	77.48	0.329	0.74	H1
2	NKI-RS	54	215	65.48	0.453	0.417	H3
3	1000FCP	78	781	24.2	0.58	0.795	H1
3	SRPBS	138	568	3.11	0.788	0.853	H1
3	camCAN	90	490	32.52	-0.205	-0.448	H3
3	NKI-RS	64	230	45.12	-0.176	-0.161	H3

While quantitative ML analysis of how brain connectivity dynamics are modulated by aging is important, it is also necessary to be cognizant of the features that are input to the predictive algorithm. The Pearson CC has been used as a measure of the degree and direction of the relationship–i.e. connectivity–among pairs of ROIs. This measure and its transformations are prevalent in rsfMRI works that investigate the effects of aging on CPs. Drawing upon several recent studies, in [27] task-evoked and rsfMRI were collected from subjects spanning 20 to 93 years. The connectivity comparisons concluded that large-scale brain organization was maintained during aging, but parcellation differences existed among age groups and increased with larger differences in age. Conversely, the resting and task fMRI data analyzed in [28] led the authors to conclude that FC was lower in older adults. Correlational analysis in [29] also noted that aging led to a decrease in strong FC across the brain. The decrease in modularity was quantified by more uniform activity patterns among the ROIs of the older subjects. The more diffuse and fewer strongly correlated functional connections with older age has been referred to as functional dedifferentiation [30–32]. We also note a definitive change in FC with increasing age that is more significant in the latter decades of the subjects. Two of the datasets show the strength of the connections to decrease (supporting functional dedifferentiation) while contrary results were noted with the SRPBS cohort. The general decrease in the mean number of significant CC values with increasing age is consistent with several prior reports of the modularity in functional networks decreasing with aging [11, 29, 33, 34]. A recent work found mostly weakened connectivity with aging among subjects, but an increase in several cases [8]. The authors attribute the increases to the within network FC of several prominent systems such as the visual and somatomotor. In [35] a four-year longitudinal analysis of N = 16 elderly subjects (69+ y.o. at the start of the study) was undertaken. The results revealed a decrease in FC in two of the three considered brain networks. Such findings are interesting in accommodating the non-unanimous results noted here when considering three distinct datasets.

The BOLD fMRI signal has been processed to quantify FC in assessing a large scale of phenotypes studied in resting and task-based studies. Surprisingly, much less attention has been given to its inter-subject variability. In this work the variability in FC was studied by computing the s.d. at various decades. An inverted U-shape was noted in quantifying the variability in two of the three datasets where the middle-aged-to-elder subjects—i.e. 51–60 y.o.—showed the highest variability. The findings point toward brain aging being a complex process where alterations in cohorts do not simply increase, decrease, or remain constant across the decades of life. The peak in variability during middle age presents intriguing possibilities as far as peoples’ lifestyle, occupation, and perhaps their personality being the most different during that juncture. Interestingly, the peak occurred at different junctures within the middle-to-elder range for male and female subjects.

Another largely neglected facet of connectivity-based fMRI analysis is the consideration of anticorrelations. While we note that the relative change of significant anticorrelations during aging generally follow what was seen for the positive correlations, there were discrepancies. Perhaps more importantly, including the negative correlations in a tally of the connectivity changes does lead to altered or new findings. With the 1000FCP subjects, if one was to restrict attention to the canonical positive connections, then a significant decrease in connectivity would be seen between the two earliest decades of the considered ages. However, incorporating the anticorrelations into the total indicates that the change is not significant. Additionally, for the 1000FCP data, the loss of FC in the male subjects for the 81+ y.o. group was not deemed significant when accounting for only the positive correlations (or the anticorrelations)–yet, the aggregate account shows statistical significance. A similar result occurred for the oldest group of male SRPBS subjects. Namely, counting the standard (positive) correlations showed no significant alteration in the number of functional connections, whereas the inclusion of anticorrelations indicated that the FC of 71+ y.o. males is modulated in comparison to the reference young group. This reveals that when assessing FC, an aggregate connectivity measure should be considered rather than solely positive correlations.

Human and animal studies are increasingly becoming cognizant of the differences between the sexes and performing separate analyses. Thus, the attainment of trajectories of healthy brain aging in males and females is important. Repeating the per-decade FC analysis for male and female subjects provided several findings. First, the changes in connectivity with aging were similar between males and females in the three datasets. Second, the modulation in the dynamics were more pronounced (i.e. statistically significant) for males. Such a result would be important to take into account when assessing deviations from a baseline in a male versus a female. Perhaps most importantly, the results reveal that the modulation in FC with age is not as prominent when male and female subjects are considered separately rather than together. This accentuates the prudence of separate sex analyses in age-related studies across a population. The inter-subject variability in FC among the sexes also yielded interesting results. Although the peak variabilities occurred at different junctures in the age spectrum between males and females, they occurred in the middle-to-elder range. This result, however, did not hold in one dataset where the youngest group of males exhibited the largest variance. Such discrepancy highlights the general difficulty in attaining consensus across datasets in light of the brain being a nonlinear stochastic system while aging is a complex process. It also warrants the requirement of further scrutiny via additional datasets, methodologies, and computation.

The datasets considered in this work share similarities as well as differences. The 1000FCP subjects spanned 17 centers necessitating an investigation of site effects. The monotonicity and change in FC analyses were repeated on subjects from the NKI-RS center to investigate if the trends noted with all subjects coincide with those seen for the largest constituent center. Interval 1 results on the presence and degree of monotonicity coincided with those attained for 1000FCP. The interval 2 findings also agreed on the absence of a monotonic relationship. Contrarily, the interval 3 conclusions were different because the NKI-RS analysis did not reflect a monotonic relationship. Aside from possible site effects, such deviation may be due to the single-site dataset providing a fewer number of subjects in the ML training set as well as in the test set. The change in FC analysis conducted on the NKI-RS subjects provided results that were very similar to the aggregate results. In the constituent and the 1000FCP, there was an increase in variability among middle age to elder subjects, and a reduction in FC variability for the two oldest decades. The trends noted for the positive and negative correlations also coincided. Furthermore, the sex differences in FC that were observed in the NKI-RS subjects agreed with those of the collective dataset. In summary, the results are suggestive of site effects not being prominent in the results presented for the 1000FCP recordings. Nevertheless, the use of harmonization techniques [36, 37] is a future avenue for further assessing to what degree the presented results may be affected.

The SRPBS and camCAN studies maintained a constant protocol with respect to all subjects having their eyes open and closed, respectively, during the recordings. This was not the case with the 1000FCP dataset (S1 Table). It is not unusual for rsfMRI investigations to consider a mixture of subjects of different arousal states [11, 38]. Nevertheless, we consider the monotonicity and quantification of FC separately for the 1000FCP recordings where subjects had their eyes open. The conclusions of the monotonicity analysis when subjects had their eyes open (N = 543) generally agreed with those attained with the aggregated subjects (S2 Fig). The hypotheses derived from interval 1 and interval 2 agree between the two studies. However, the interval 3 results indicate the absence of a monotonic relationship between the chronological age and the percentage of subjects classified as aged when considering only subjects with eyes open during recordings (S2C Fig). The findings pertaining to the change in FC across decades were consistent when considering all 1000FCP subjects and separately studying the subjects with eyes open (S2D Fig).

The study of FC alterations with aging was repeated with thresholds of |ρ = 0.45| and |ρ = 0.7| for declaring connections (S3 and S4 Figs). Scrutiny with lower and higher thresholds aims to expurgate spurious relationships that would arise with noise-dominated signals (i.e. low SNR recordings). With the decreased value of |ρ = 0.45|, the per-dataset trends in the mean and variability of the number of FCs across the age spectrum matched what was reported in Fig 6. There were naturally a larger number of FCs per age group with the lower threshold. Similarly, although a higher threshold lowered the number of FCs in every age group, the trends in the mean and variability were consistent to those observed in Fig 6. The results are reassuring by reproducing the findings despite less stringent as well as more conservative threshold values from what we initially considered. Nevertheless, more detailed analysis of the FC dependency during aging on the SNR and threshold values is an avenue of future research. There is an additional caveat of the number of ROIs not being constant among the considered datasets. However, it is not uncommon in fMRI studies that examine multiple datasets to assess BOLD differences with different ROI numbers and selection schemes [39–41]. For instance, the authors of [42] compared age prediction results with 419 ROIs from HCP subjects and 55 ROIs from UK Biobank subjects. Despite the ML pipeline ensuring that the same number of young and aged subjects are used to train an SVM, there is an unequal number of subjects considered across the age groups in the datasets. This is a consequence of the subject imbalance between ages that exists in neuroimaging studies where age is a predictive variable and spans a broad range. Rather than being a hinderance, this is standard among related works [8, 43]. The number of data points across the subjects at different recording centers was also not constant. This is consistent with related works such as [11, 44]. A recent work evaluated brain age prediction accuracy for varying number of time points and observed improvements with the number of time points [45]. However, the increases were reported as being relatively small. Lastly, it should be noted that more sophisticated and computationally demanding algorithmic techniques such as DL may provide more accurate prediction results and reveal FC properties in any one of the considered datasets. However, the techniques may also overfit a different dataset to provide marginal predictive capability and obscure FC characteristics by not generalizing. An SVM classifier was considered in this work due to its simplicity, general robustness, and interpretability. A comparative study with more advanced methods is a future avenue of investigation.

## Supporting information

S1 Fig Brain age monotonicity without application of a sliding window. The results of the brain age monotonicity study for intervals 1, 2, and 3 without application of a 5-year sliding window. To test for a trend, a linear regression was fit to the data with the intercept β_0_ and slope β_1_. A) For 1000FCP subjects, in interval 1 we have β_0_ = 31.49, β_1_ = 0.971. The Spearman correlation of 0.549 computed among the percentages of subjects classified as aged and the ages indicates a moderate degree of monotonicity between the two quantities. In interval 2 we attain β_0_ = 70.94, β_1_ = -0.108 indicating little change in the classification of increasingly older brains as aged. The Spearman correlation of -0.053 reflects a non-monotonic relationship among the ages and the percentage of subjects classified as aged. For interval 3, the values β_0_ = 24.33, β_1_ = 0.568, and the Spearman correlation of 0.404 signifies a gradual increase and small degree of monotonicity. B) When considering the SRPBS subjects, in interval 1 we have β_0_ = 6.647, β_1_ = 2.749. The Spearman correlation of 0.818 computed among the percentages of subjects classified as aged and the ages demonstrates monotonicity between the two quantities. In interval 2 we attain β_0_ = 82.49, β_1_ = 0.054 indicating little change in the classification of increasingly older brains as aged. The Spearman correlation of 0.108 reflects a non-monotonic relationship among the subjects’ ages and the percentage labeled as aged. For interval 3, the values β_0_ = 3.48, β_1_ = 0.748, and the Spearman correlation of 0.544 indicates an increase and moderate degree of monotonicity. C) For the camCAN dataset, in interval 1 we have β_0_ = 0, β_1_ = 2.254. The Spearman correlation of 0.9027 computed among the percentages of subjects classified as aged and the subject ages signifies a high degree of monotonicity between the two quantities. In interval 2 we attain β_0_ = 76.42, β_1_ = 0.3706 demonstrating little change in the classification of increasingly older brains as aged. The Spearman correlation of 0.386 reflects a non-monotonic relationship. For interval 3, the values β_0_ = 32.301, β_1_ = -0.1968, and the Spearman correlation of -0.118 indicate a gradual decrease and non-monotonicity in the number of subjects classified as aged with increasing biological age. D) With the NKI-RS subjects, in interval 1 we have β_0_ = 39.71, β_1_ = 0.858. The Spearman correlation of 0.396 demonstrates small to no monotonicity between the percentages of subjects classified as aged and the ages. In interval 2 we attain β_0_ = 64.78, β_1_ = 0.496 which indicates a gradual increase in the classification of increasingly older brains as aged. The Spearman correlation of 0.371 reflects an absence of monotonicity among the subjects’ ages and the percentage labeled as aged. For interval 3, the values β_0_ = 45.75, β_1_ = -0.206, and the Spearman correlation of -0.112 indicates a non-monotonic but decreasing relationship.
(TIFF)

S2 Fig Findings for subjects with eyes open during the rsfMRI recordings. The results of the brain age monotonicity and change in FC studies for subjects in the 1000FCP dataset that had eyes open during rsfMRI recording. For the monotonicity study, the histograms show the number of subjects classified as young and aged at every considered age. A 5-year sliding window was applied and a linear regression fit to the results with the intercept β_0_ and slope β_1_. A) The analysis consists of a machine trained on the youngest and oldest groups and presented with test data of intermediate-aged subjects. In interval 1 we have β_0_ = 22.71, β_1_ = 1.34. The Spearman correlation of 0.661 computed among the percentages of subjects classified as aged and the ages indicates a moderate degree of monotonicity. B) A machine was trained on the two youngest groups of subjects and then provided with recordings from subjects 41+ y.o. (interval 2). The values β_0_ = 54.2, β_1_ = 0.76, and a Spearman correlation of 0.325 indicate an absence of monotonicity. C) A machine was trained on subjects from the two oldest age groups prior to being presented with the task of classifying younger subjects (21–60 y.o.) in interval 3. The linear fit values β_0_ = 34.32, β_1_ = 0.25, and Spearman correlation of 0.047 signify a non-monotonic relationship between chronological age and the percentage of subjects classified as aged. D) The change in FC analysis consisted of the mean FC computed by averaging across subjects the number of times that the Pearson CCs from the connectivity matrix exceeded |*ρ*| = 0.6. The s.d. of the number of functional connections is shown to study the inter-subject variability across the age spectrum.
(TIFF)

S3 Fig Analysis of FC changes with threshold |*ρ*| = 0.45. A study of the change in FC for subjects across decades from the A) 1000FCP, B) SRPBS, and C) camCAN datasets. A threshold |*ρ*| = 0.45 was used to determine if the Pearson CCs from the connectivity matrix constituted a FC. The mean FC was computed by averaging the FC values across the subjects in each age group. A bifurcation of the FC to positive and negative (i.e. anticorrelations) directions is also shown. Two-sample t-tests were used to assess the significance of the change in FC with the youngest age group taken as the reference. A p-value of 0.05 is the threshold for statistical significance in the pairwise comparisons of positive, negative, and total FC values. The s.d. of the number of functional connections is illustrated to study the inter-subject variability across the age spectrum.
(TIFF)

S4 Fig Analysis of FC changes with threshold |*ρ*| = 0.7. A study of the change in FC for subjects across decades from the A) 1000FCP, B) SRPBS, and C) camCAN datasets. A threshold |*ρ*| = 0.7 was used to determine if the Pearson CCs from the connectivity matrix constituted a FC. The mean FC was computed by averaging the FC values across the subjects in each age group. A bifurcation of the FC to positive and negative (i.e. anticorrelations) directions is also shown. Two-sample t-tests were used to assess the significance of the change in FC with the youngest age group taken as the reference. A p-value of 0.05 is the threshold for statistical significance in the pairwise comparisons of positive, negative, and total FC values. The s.d. of the number of functional connections is illustrated to study the inter-subject variability across the age spectrum.
(TIFF)

S1 Table 1000FCP subjects with eyes open or closed. A listing of the subjects from the 1000FCP with eyes open (N = 543) or closed (N = 197) during the rsfMRI recording.
(TIFF)
