# Supplementary figures and images for: Brain age monotonicity and functional connectivity differences of healthy subjects
Source: PLoS One. 2024 May 30;19(5):e0300720. doi: 10.1371/journal.pone.0300720 (PMC11139261; doi:10.1371/journal.pone.0300720)

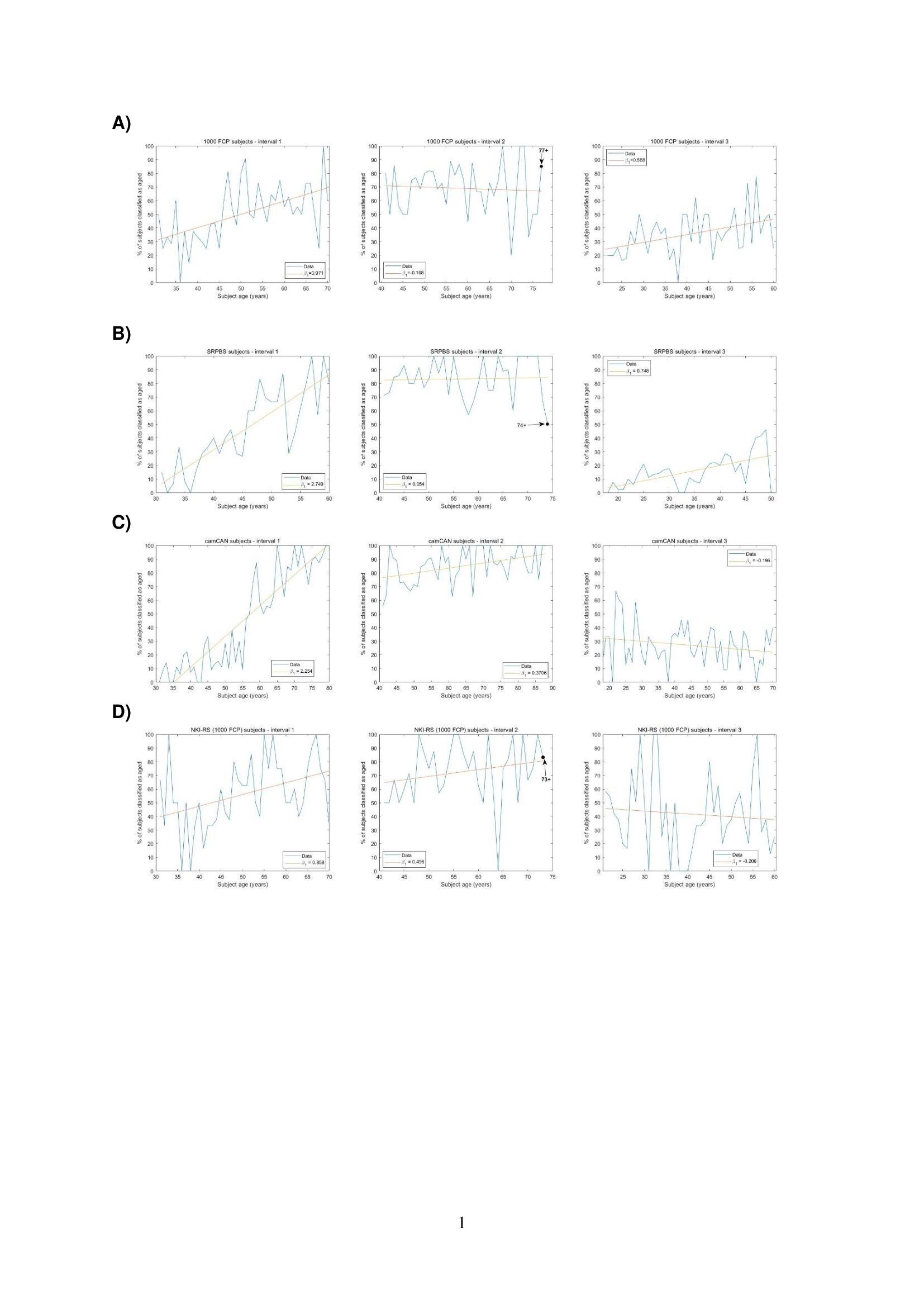

Supplement: S1 Fig — The results of the brain age monotonicity study for intervals 1, 2, and 3 without application of a 5-year sliding window. To test for a trend, a linear regression was fit to the data with the intercept β0 and slope β1. A) For 1000FCP subjects, in interval 1 we have β0 = 31.49, β1 = 0.971. The Spearman correlation of 0.549 computed among the percentages of subjects classified as aged and the ages indicates a moderate degree of monotonicity between the two quantities. In interval 2 we attain β0 = 70.94, β1 = -0.108 indicating little change in the classification of increasingly older brains as aged. The Spearman correlation of -0.053 reflects a non-monotonic relationship among the ages and the percentage of subjects classified as aged. For interval 3, the values β0 = 24.33, β1 = 0.568, and the Spearman correlation of 0.404 signifies a gradual increase and small degree of monotonicity. B) When considering the SRPBS subjects, in interval 1 we have β0 = 6.647, β1 = 2.749. The Spearman correlation of 0.818 computed among the percentages of subjects classified as aged and the ages demonstrates monotonicity between the two quantities. In interval 2 we attain β0 = 82.49, β1 = 0.054 indicating little change in the classification of increasingly older brains as aged. The Spearman correlation of 0.108 reflects a non-monotonic relationship among the subjects’ ages and the percentage labeled as aged. For interval 3, the values β0 = 3.48, β1 = 0.748, and the Spearman correlation of 0.544 indicates an increase and moderate degree of monotonicity. C) For the camCAN dataset, in interval 1 we have β0 = 0, β1 = 2.254. The Spearman correlation of 0.9027 computed among the percentages of subjects classified as aged and the subject ages signifies a high degree of monotonicity between the two quantities. In interval 2 we attain β0 = 76.42, β1 = 0.3706 demonstrating little change in the classification of increasingly older brains as aged. The Spearman correlation of 0.386 reflects a non-mono [file pone.0300720.s001.tiff]

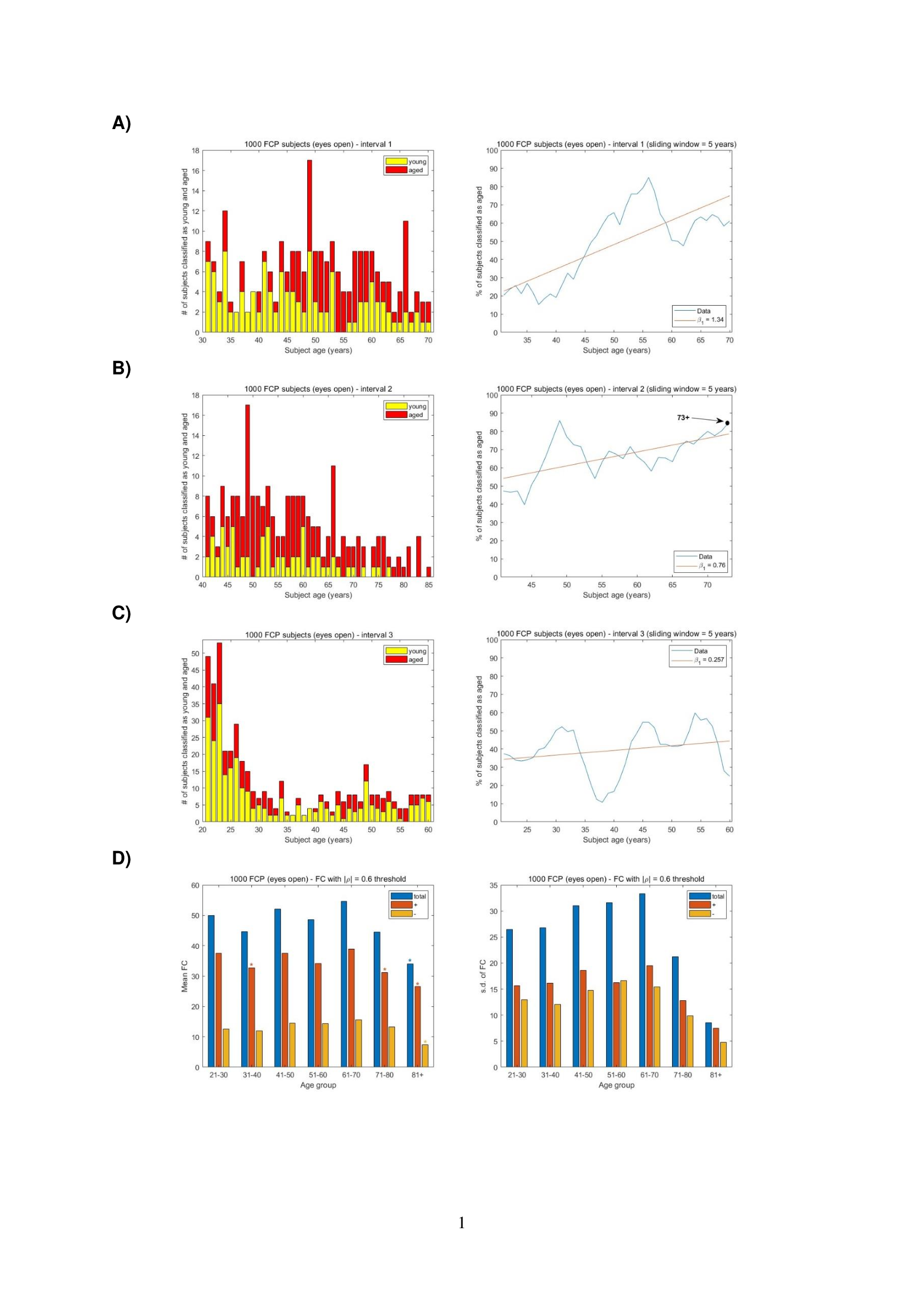

Supplement: S2 Fig — The results of the brain age monotonicity and change in FC studies for subjects in the 1000FCP dataset that had eyes open during rsfMRI recording. For the monotonicity study, the histograms show the number of subjects classified as young and aged at every considered age. A 5-year sliding window was applied and a linear regression fit to the results with the intercept β0 and slope β1. A) The analysis consists of a machine trained on the youngest and oldest groups and presented with test data of intermediate-aged subjects. In interval 1 we have β0 = 22.71, β1 = 1.34. The Spearman correlation of 0.661 computed among the percentages of subjects classified as aged and the ages indicates a moderate degree of monotonicity. B) A machine was trained on the two youngest groups of subjects and then provided with recordings from subjects 41+ y.o. (interval 2). The values β0 = 54.2, β1 = 0.76, and a Spearman correlation of 0.325 indicate an absence of monotonicity. C) A machine was trained on subjects from the two oldest age groups prior to being presented with the task of classifying younger subjects (21–60 y.o.) in interval 3. The linear fit values β0 = 34.32, β1 = 0.25, and Spearman correlation of 0.047 signify a non-monotonic relationship between chronological age and the percentage of subjects classified as aged. D) The change in FC analysis consisted of the mean FC computed by averaging across subjects the number of times that the Pearson CCs from the connectivity matrix exceeded |ρ| = 0.6. The s.d. of the number of functional connections is shown to study the inter-subject variability across the age spectrum. (TIFF) [file pone.0300720.s002.tiff]

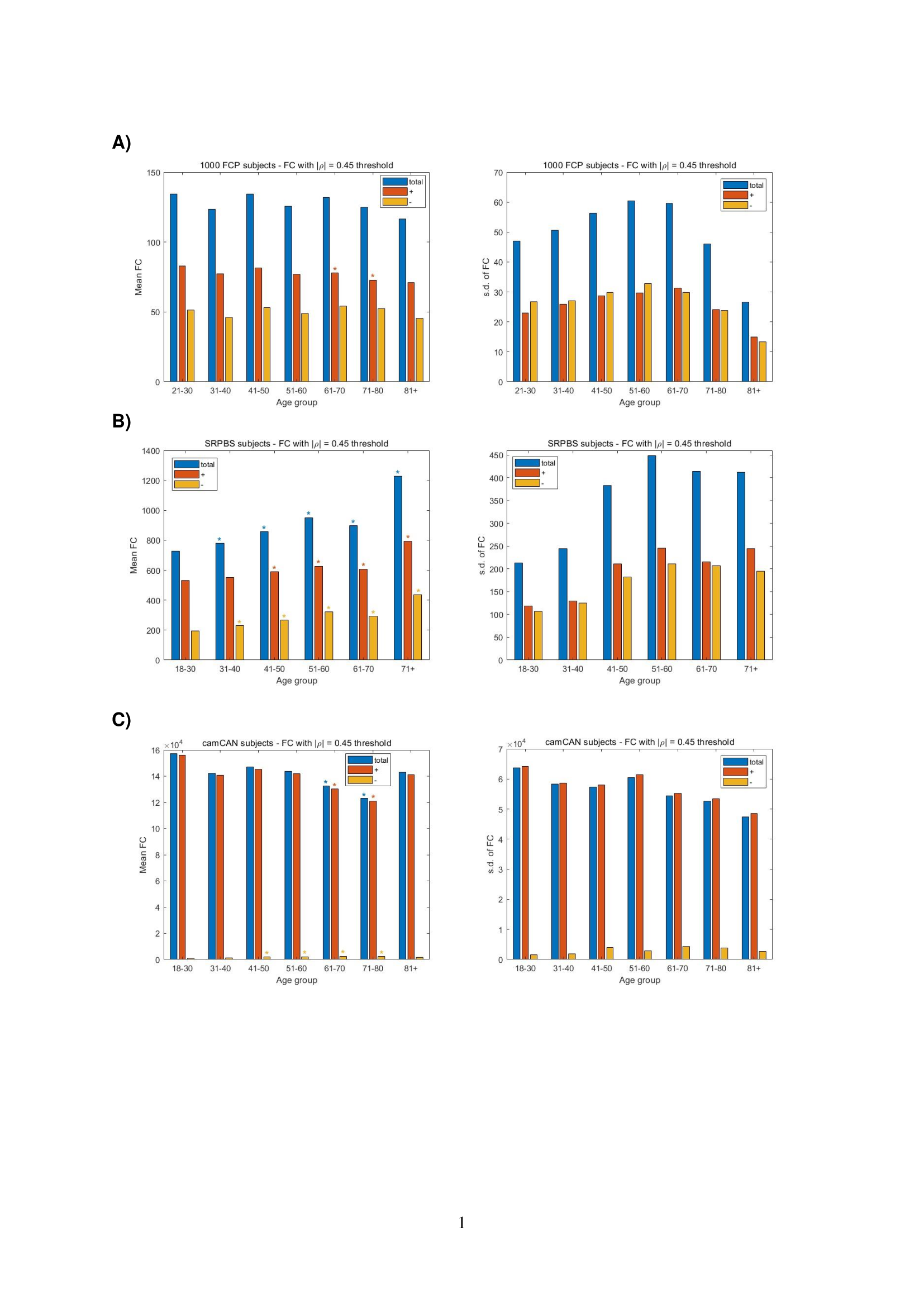

Supplement: S3 Fig — A study of the change in FC for subjects across decades from the A) 1000FCP, B) SRPBS, and C) camCAN datasets. A threshold |ρ| = 0.45 was used to determine if the Pearson CCs from the connectivity matrix constituted a FC. The mean FC was computed by averaging the FC values across the subjects in each age group. A bifurcation of the FC to positive and negative (i.e. anticorrelations) directions is also shown. Two-sample t-tests were used to assess the significance of the change in FC with the youngest age group taken as the reference. A p-value of 0.05 is the threshold for statistical significance in the pairwise comparisons of positive, negative, and total FC values. The s.d. of the number of functional connections is illustrated to study the inter-subject variability across the age spectrum. (TIFF) [file pone.0300720.s003.tiff]

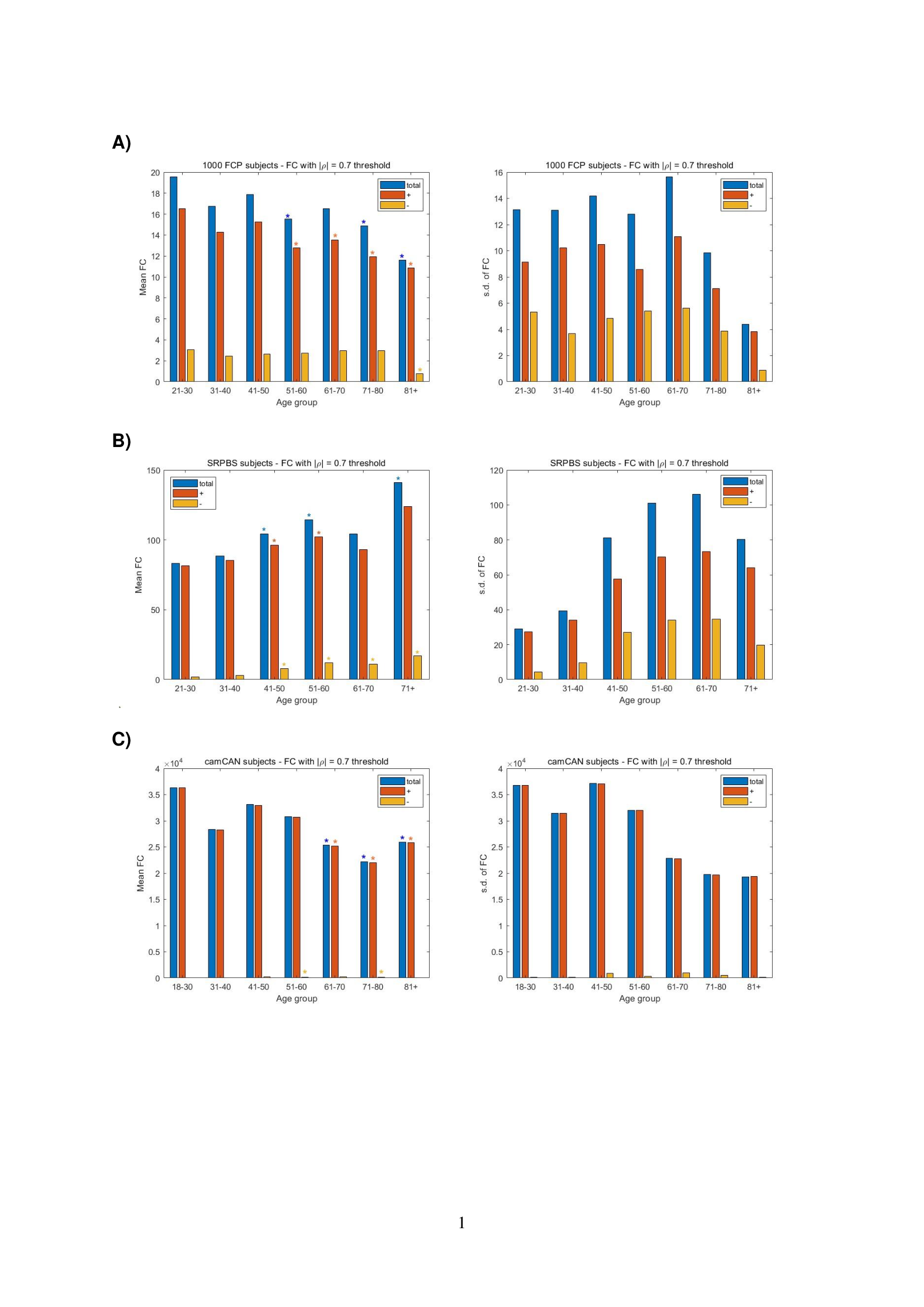

Supplement: S4 Fig — A study of the change in FC for subjects across decades from the A) 1000FCP, B) SRPBS, and C) camCAN datasets. A threshold |ρ| = 0.7 was used to determine if the Pearson CCs from the connectivity matrix constituted a FC. The mean FC was computed by averaging the FC values across the subjects in each age group. A bifurcation of the FC to positive and negative (i.e. anticorrelations) directions is also shown. Two-sample t-tests were used to assess the significance of the change in FC with the youngest age group taken as the reference. A p-value of 0.05 is the threshold for statistical significance in the pairwise comparisons of positive, negative, and total FC values. The s.d. of the number of functional connections is illustrated to study the inter-subject variability across the age spectrum. (TIFF) [file pone.0300720.s004.tiff]
